# Combined Effects of Thrombosis Pathway Gene Variants Predict Cardiovascular Events

**DOI:** 10.1371/journal.pgen.0030120

**Published:** 2007-07-27

**Authors:** Kirsi Auro, Mervi Alanne, Kati Kristiansson, Kaisa Silander, Kari Kuulasmaa, Veikko Salomaa, Leena Peltonen, Markus Perola

**Affiliations:** 1 Department of Molecular Medicine, National Public Health Institute, Helsinki, Finland; 2 Department of Health Promotion and Chronic Disease Prevention, National Public Health Institute, Helsinki, Finland; 3 Department of Medical Genetics, University of Helsinki, Helsinki, Finland; 4 The Broad Institute of MIT and Harvard, Boston, Massachusetts, United States of America; University of Alabama at Birmingham, United States of America

## Abstract

The genetic background of complex diseases is proposed to consist of several low-penetrance risk loci. Addressing this complexity likely requires both large sample size and simultaneous analysis of different predisposing variants. We investigated the role of four thrombosis genes: coagulation factor V *(F5),* intercellular adhesion molecule 1 *(ICAM1),* protein C *(PROC),* and thrombomodulin *(THBD)* in cardiovascular diseases. Single allelic gene variants and their pair-wise combinations were analyzed in two independently sampled population cohorts from Finland. From among 14,140 FINRISK participants (FINRISK-92, *n* = 5,999 and FINRISK-97, *n* = 8,141), we selected for genotyping a sample of 2,222, including 528 incident cardiovascular disease (CVD) cases and random subcohorts totaling 786. To cover all known common haplotypes (>10%), 54 single nucleotide polymorphisms (SNPs) were genotyped. Classification-tree analysis identified 11 SNPs that were further analyzed in Cox's proportional hazard model as single variants and pair-wise combinations. Multiple testing was controlled by use of two independent cohorts and with false-discovery rate. Several CVD risk variants were identified: In women, the combination of *F5 rs7542281* × *THBD rs1042580,* together with three single *F5* SNPs, was associated with CVD events. Among men, *PROC rs1041296,* when combined with either *ICAM1 rs5030341* or *F5 rs2269648,* was associated with total mortality. As a single variant, *PROC rs1401296,* together with the *F5* Leiden mutation, was associated with ischemic stroke events. Our strategy to combine the classification-tree analysis with more traditional genetic models was successful in identifying SNPs—acting either in combination or as single variants—predisposing to CVD, and produced consistent results in two independent cohorts. These results suggest that variants in these four thrombosis genes contribute to arterial cardiovascular events at population level.

## Author Summary

The genetic background of cardiovascular diseases is still largely unknown. As in other complex diseases, the genetic risk is thought to consist of several genetic variants and their possible interactions. Elucidation of the genetic component of any complex trait most likely requires simultaneous examination of various genes in large and well-characterized study samples. Our study explores the role in cardiovascular disease of four thrombosis genes: coagulation factor V, intercellular adhesion molecule 1, protein C, and thrombomodulin. These genes form a physiological pathway that is part of the coagulation cascade. Their defects, such as the Leiden mutation in factor V gene, predispose to venous thrombosis, making them tempting candidates for involvement with cardiovascular events. Given the interactions of these genes at the molecular level, a logical way to explore their genetic effects is simultanous rather than gene-specific analysis of all the four genes, aiming to illustrate their possible interplay. The study setting combines classification trees with classic genetic analyses and covers all known common variants of the genes. The results imply that the variants in these four genes contribute to disease risk either alone or in various allelic combinations.

## Introduction

The genetic basis of complex diseases like coronary heart disease and ischemic stroke probably consists of several predisposing risk factors that can interact with environmental factors to produce the disease phenotype. To address such polygenic structure is a challenge likely requiring simultaneous analysis of several risk factors, including genetic variants, in large study samples rich in phenotypes. Gene–gene and gene–environment interaction studies have recently attempted to answer this challenge by analyzing the interacting relations of putative risk loci [1–5]. The majority of these studies, however, use two to three genetic markers, thus failing to address the physiological entities or the underlying complex genetic profiles.

In a physiological clotting cascade, binding of thrombin to its receptor, thrombomodulin (THBD), activates protein C (PROC). Activated protein C cleaves coagulation factor V (F5) [6], leading to fibrinolysis. Genetic variation in the cascade genes predisposes to increased clotting, the best known example being activated protein C resistance caused by the factor V Leiden mutation [7]. Several case reports describe PROC deficiency in patients with arterial thrombosis [8,9]. Activated protein C may also play a neuroprotective role in ischemic stroke [10,11]. THBD and intercellular adhesion molecule 1 (ICAM1) are markers for endothelial activation and damage [12]. Low concentrations of soluble THBD, especially when present along with elevated soluble ICAM1, predispose to cardiovascular disease (CVD) events [13,14]. Whether *THBD* gene variants act as independent CVD risk factors remains unclear [15].

Studies on these four genes have mainly concentrated on a few, often rare, functional polymorphisms within the individual genes [16–19]. We hypothesize that analyzing allelic variants of several genes encoding components of the same physiological cascade will prove to be a more powerful approach to shedding light on CVD risk mechanisms than are studies on single candidates. Analyses comprising several genes belonging to the same pathway may reveal cumulative allelic effects. When acting together, these gene variants may affect the disease risk more profoundly than do the single predisposing variants. We covered common variants of *F5, ICAM1, PROC*, and *THBD* genes and assessed their role in CVD in two prospective and independently sampled population cohorts of Finns. To address the single gene variants and their interplay in cardiovascular traits, we chose a two-step strategy. We first aimed to identify the variants contributing most to the CVD risk in our study sample using classification-trees. Second, based on these analyses, we studied a subset of the most important SNPs by classical genetic analyses, first as independent markers and then as pair-wise combinations as deviation from the multiplicative model of genetic interaction. Our study identified several variants predisposing to CVD as single or cooperating markers.

## Materials and Methods

### Study Samples

We utilized two large, independently sampled, and prospectively followed population cohorts from Finland, FINRISK-92 (*n* = 5,999, follow-up 1992–2001) and FINRISK-97 (*n* = 8,141, follow-up 1997–2003). Subjects for genotyping were selected according to a case-cohort design from among 14,140 FINRISK participants. As cases in this study, we consider those with an incident coronary event (coronary heart disease [CHD], *n* = 401) or ischemic stroke (*n* = 149) during follow-up and did not have acute coronary events or strokes before the baseline examination, and everyone deceased from any reason during follow-up (*n* = 610). In addition, individuals having experienced cardiovascular events at baseline were genotyped, but baseline CVD was not utilized as an endpoint in this study. International Classification of Diseases (ICD) −9 and −10 codes for fatal coronary events were 410–414 and 798, and I20–I25, I46, R96, R98, R99, and for nonfatal coronary events 410–411 (ICD-9) and I20.0 and I21–I22 (ICD-10). For fatal and nonfatal ischemic strokes, ICD-9 codes 433 (excluding 4330X, 4331X, and 4339X of the Finnish modification of ICD-9), 434 (excluding 4349X), and ICD-10 code I63 were used. The ICD-10 revision has been used in Finland since January 1996. We will denote the combination of coronary event and ischemic stroke endpoints by CVD. Because 21 individuals had both an incident coronary event and an incident ischemic stroke, the total number of individuals having incident CVD events was 528. Of the 610 deceased, 138 also had an incident CVD event. Random subcohorts, selected with age-dependent probabilities, were drawn from the original cohorts to represent the general study populations (FINRISK-92 *n* = 400, FINRISK-97 *n* = 386). Thus, the subcohorts also included individuals having CVD events at baseline (*n* = 88) or during follow-up (*n* = 72). This selection yielded a study sample of 2,222 individuals for genotyping (Table 1). Information on the traditional CVD risk factors (serum cholesterol, triglyceride (TG), and C-reactive protein (CRP) levels, blood pressure, anthropomorphic measures, smoking, medication, disease, and family history) as well as whole blood samples for DNA were collected at baseline. The study cohorts have been described in detail previously [15,20] and at http://www.ktl.fi/publications/morgam/cohorts/index.html. The Ethics Committee of the National Public Health Institute of Finland approved the study.

**Table 1:**
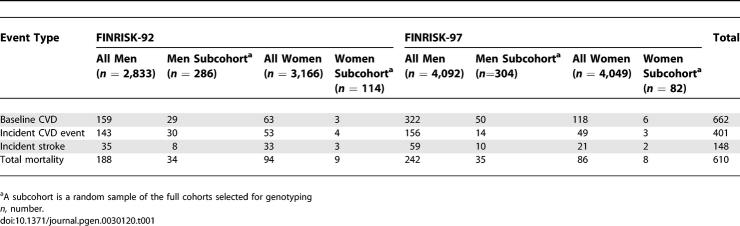
Characteristics of the Study Cohorts: Number of Cardiovascular Events

### Genotyping Strategy

To cover all known common (frequency >10%) variants in the four thrombosis genes, 54 single nucleotide polymorphisms (SNPs) were selected: 24 SNPs in *F5*, nine in *ICAM1*, seven in *PROC*, and 14 in *THBD* [15]. This selection was based on haplotype information in the SeattleSNPs variation discovery resource [21]. The *F5* Leiden mutation *(rs6025)* was included based on the literature. Haplotype structures in the four genes were analyzed with Haploview3.2 [22]. For any two SNPs in tight linkage disequilibrium (LD, *r* ^2^ > 0.8) with each other, only one was included in further analyses. Because SNP selection was based on haplotype information, haplotype analyses were excluded. Baseline CVD cases were utilized in LD calculations, but had to be excluded from Cox's proportional hazard model due to the unknown time of the event measured (before the study began). Thus, the baseline cases were excluded from all statistical analyses to achieve a similar study sample used in all the analyses. *Rs3216183* was genotyped with TaqMan (Roche Molecular Systems, http://www.roche.com/) and the remaining 53 SNPs with Sequenom MassARRAY (Sequenom, http://www.sequenom.com/) with 10 ng of DNA and hME chemistry. Primer information and assay conditions are available from the authors upon request. DNA was extracted by standard protocols [23] from whole blood stored at −20 °C. *ICAM1* SNP *r5030380* was excluded due to technical difficulties. The samples with low DNA yield (*n* = 100) were whole-genome amplified before genotyping [24]. Before genotyping the FINRISK samples, the Mendelian inheritance of each SNP was checked in a Finnish sample of 60 mother, father, and child trios. Mendel check revealed no errors. The FINRISK genotypic sample included 2% open and 5% blinded duplicates. Genotyping was performed as part of the MORGAM and GenomEUtwin Projects (http://www.genomeutwin.org/) [25]. The genotyping error rate was <1/400 in blind duplicate comparisons. The genotyping success rate was 93% or higher for each SNP. Together, these quality-control -procedures suggested very high genotyping quality.

### Statistical Analyses

Statistical analyses were performed in three stages: (1) with AnswerTree3.0 (SPSS, http://www.spss.com/) to discover possible co-appearance of allelic variants as risk definers and to select the most significant SNPs for further analyses, and (2) with SAS v8.2 for Windows (SAS, http://www.sas.com/) to estimate allele frequencies, to discover SNPs influencing the traditional CVD risk factors, and to estimate hazard ratios (HR) of the SNP alleles with Cox's proportional hazards model [26]. (3) Finally, to estimate whether the SNPs chosen at stage 1 were the informative ones required a separate sensitivity analysis. All SNPs were analyzed in Cox's proportional hazards, coding the SNPs as 1–0 assuming dominant inheritance.

AnswerTree is a classification-tree algorithm that attempts to find a variable and a cutpoint for the variable to split a dataset to best predict disease outcome. This split is called the root node. The algorithm is then applied to the resulting subgroups of the dataset accordingly, and the process is continued to form a tree. In our application of the algorithm, SNPs were coded as 0 (minor allele carriers) to 1 (major allele homozygotes) assuming dominant inheritance and analyzed together with the traditional risk factors, using incident CVD events as the endpoint. Of the possible algorithms we used exhaustive chi-squared automatic interaction detector (CHAID), which allows more than two sister nodes in a split if necessary and thus was thought to model the biological risk caused by the phenotypic risk factors, for example cholesterol values, better than a strictly bifurcated split, and moreover, it allows control of a number of steps in the tree: To keep the tree structure simple and to discover combinations of predisposing factors affecting relatively large groups of individuals, we set the minimum root node size at 20 and the minimum end node size at 10. Maximum branch level in the trees was limited to five. The significance of all splits was calculated as χ^2^

To search for all well-supported splits and to reduce the extent to which the root node in the overall analysis may obscure other important splits we decided, instead of growing a single tree, to grow a forest of trees: a collection of classification trees achieved by bootstrap techniques using random subsamples and other criteria. In order to grow the forest, we took ten random subsamples of 60% of the men and ten random subsamples of 60% of the women of the combination of the FINRISK-92 and −97 cohorts. First, one tree was grown for each subsample to determine the significant splits. These 20 trees included altogether 50 significant splits for men and 45 for women (Tables 1 and 2). Using these significant splits from the 20 trees as root nodes we grew a forest of 50 trees for men and 45 trees for women still utilizing the subsample data. The SNPs present in >10% of the sex-specific trees were selected for further analysis. Because the data used in the classification-tree analysis originated from a single dataset, combining the two cohorts, no cross validation among the samples was performed.

**Table 2:**
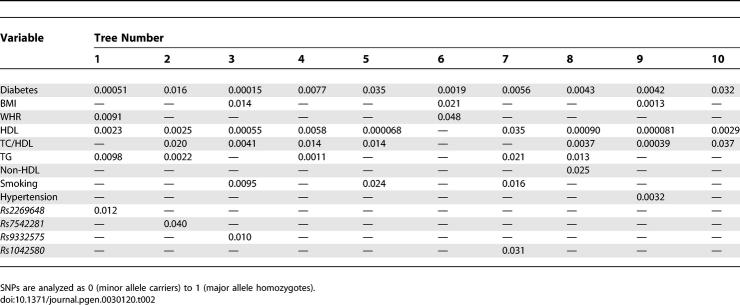
Significant Splits (Sample-Specific *p*-Value, χ^2^) Seen in Classification-Trees Grown in AnswerTree3.0 with Ten Random Female Datasets, Each Containing 60% of the Original Data

At stage 2, we first used the combined data set of FINRISK-92 and −97 cohorts to gain maximum power, but also both cohorts separately to assess the consistency of the findings. The allele distributions of the selected SNPs between the cases and the subcohort (excluding cases and baseline CVD) were compared with Pearson's χ^2^. The relation of each SNP to the common CVD risk factors (TG, total cholesterol [TC], HDL cholesterol [HDL], TC/HDL ratio, systolic and diastolic blood pressure, CRP, body mass index [BMI], and waist-to-hip ratio [WHR]) was analyzed separately for each risk factor with an age-, sex-, and cohort-adjusted general linear model. To achieve normality, HDL and CRP were log transformed. Hazard ratios for all selected SNPs and their pair-wise combinations (with interaction terms, as deviation from the multiplicative model of interaction) were calculated with Cox's proportional hazards model, with age at baseline, (sex), TC/HDL ratio, BMI, smoking, diabetes, hypertension, and CRP as covariates. Time from baseline served as the time parameter. The analysis was stratified by eastern and western Finland and additionally by cohort when combining the two datasets. Total mortality, incident coronary, incident ischemic stroke, and incident CVD (coronary and stroke events combined) events served as endpoints. We first performed sex-specific analyses and then combined men and women, under additive, dominant, and recessive inheritance models. The *F5* Leiden mutation was analyzed only with the dominant model, due to low minor allele frequency. Multiple testing was addressed by performing all analyses in the two separate cohorts, as well as with false-discovery rate (FDR). FDR was calculated within sex- and endpoint-specific groups. SNP combinations were analyzed with interaction terms as deviation from the multiplicative model (i.e., interaction HR different from the product of the independent SNP HRs) in Cox's model adjusted for the same risk factors as with the single SNPs. With interaction terms, the recessive model of inheritance was selected for a SNP if it showed significant association in the single SNP analyses, otherwise the dominant model was used to gain power. Results showing a consistent association or similar trend in the two separate study cohorts or their combination or both, and having FDR <0.1 for their combination were considered as significant.

## Results

In both cohorts and in both sexes incident CVD cases had higher total cholesterol and BMI and lower HDL cholesterol at baseline than did subcohort members free of CVD at the end of follow-up. The CVD cases also had a higher frequency of diabetes and hypertension and more often smoked than did the healthy subcohort members (Table 3). The baseline characteristics of the cases and the subcohorts have been described in detail [15,20].

**Table 3:**
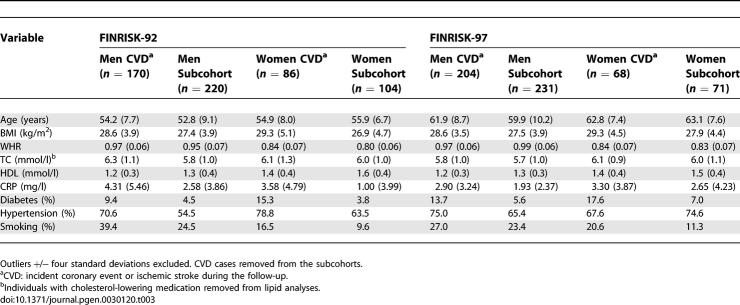
Characteristics (Mean [Standard Deviation] or %) of the Study Cohorts

Based on analyses with Haploview3.2, the LD pattern of our study sample of Finns, a population isolate known to retain higher levels of LD [27], differed from that provided by Seattle SNPs, and we excluded from the analyses four F5 SNPs (*rs3766103, rs2227245, rs6670678,* and *rs6029,* Figure S1), one *ICAM1* SNP *(rs5030352),* one *PROC* SNP *(rs2069921),* and one *THBD* SNP *(rs1042579),* all showing strong LD (*r* ^2^ ≥ 0.8) with another SNP. In addition, SNPs monomorphic in 370 Finnish individuals were excluded *(rs5030388, rs9332566, rs9332625, rs9332587, rs1046712, rs1800576, rs1800577, rs1800578, rs1800579, rs3176122,* and *rs3176121)*. The genotype frequencies of the remaining 36 SNPs (Table S2) followed Hardy–Weinberg equilibrium in the subcohorts (*p* > 0.05).

**Figure 1:**
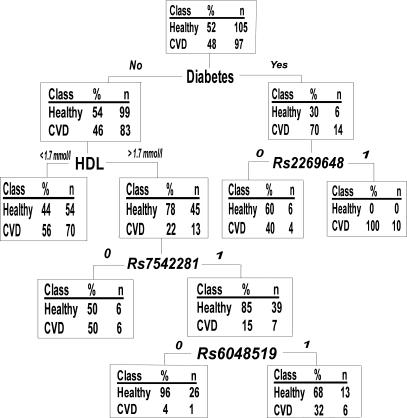
Example of a Classification-Tree in a Random Female Subsample Consisting of 60% of the Original Data SNPs are analyzed as 0 (minor allele carriers) to 1 (major allele homozygotes), modeling dominant inheritance.

We aimed to reduce the number of variants used in further analyses and to discover possible co-effects of single gene variants by classification-tree analyses. The 36 SNPs, together with the baseline phenotypic variables (smoking, TC, TG, HDL, BMI, WHR, systolic and diastolic blood pressure, diabetes status, and CRP), were analyzed with AnswerTree3.0 with incident CVD as an endpoint. We grew a forest of classification-trees using each of the significant splits of Table 2 and Table S1 as root nodes. From this 95-tree forest (50 trees for men and 45 for women), we selected all SNPs present in >10% of the trees for further analyses. This selection yielded 12 SNPs: six *F5* SNPs and two SNPs each from *THBD, PROC,* and *ICAM1*. Of the *F5* SNPs, *rs970741* was excluded due to relatively strong LD (*r* ^2^ = 0.42) with *rs2420369* (Figure S1), further reducing the SNP selection to 11 (Table 4). The rationale for this exclusion was to avoid an LD-based bias when analyzing SNP combinations. Figure 1 gives an example of a single tree grown with a random female sample. The figure also demonstrates a general pattern observed: the best splits seen with all subsamples were traditional phenotypic risk factors such as cholesterol or BMI, and the SNPs played a role in the lower branches of the trees, i.e., in subgroups of individuals specified by the phenotypic factors (Figure 1, Table 2, Table S1).

**Table 4:**
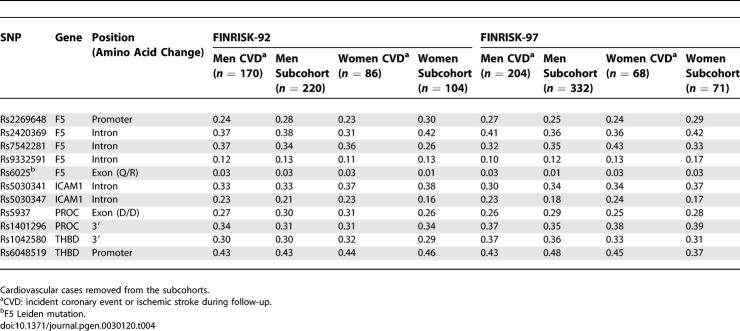
Minor Allele Frequencies of the SNPs Analyzed in Stage 2 of the Study

Next, we analyzed genotype–phenotype relationships of the 11 selected SNPs under additive, dominant, and recessive models of inheritance. The male minor allele carriers of *ICAM1 rs5030341* had significantly smaller waist-to-hip ratio (p=0.0077 for combined cohorts, data not shown) than did the non-carriers of this allele, and the CVD cases homozygotic for *F5 rs7542281* had significantly lower BMI (*p* = 0.0018 for combined cohorts, unpublished data). These observations were consistent in both cohorts.

Separate Cox's proportional hazard models were fitted for all selected 11 SNPs and their pair-wise combinations. Incident coronary, incident ischemic stroke, incident cardiovascular events, and total mortality served as endpoints. We considered only SNPs showing consistent association in the separate cohorts or their combination or both, and exceeding the FDR <10% limit for the combined cohorts as significant. With pair-wise SNP combinations, a notable deviation from the multiplicative interaction model was additionally required. The Cox's model revealed several significant CVD risk variants (Tables 3, 5, and 6). Among women, *F5* SNPs *rs7542281* was associated with incident CVD. When combined with *THBD rs1042580,* substantial deviation from multiplicative model of interaction was seen. In addition, *F5 rs2420369* was associated with incident CVD events. In men, *PROC rs1401296* was associated with total mortality when combined with *ICAM1 rs5030341* or with *F5 rs2269648*. *ICAM1 rs5030347* was associated with total mortality as a single variant.

**Table 5:**
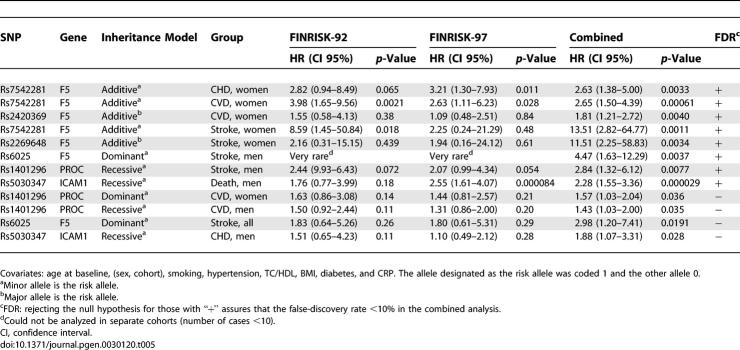
Association of Single SNPs with the Endpoints in Time-to-Event Analysis

**Figure 2:**
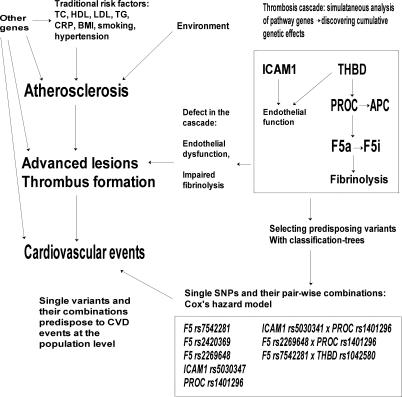
Schematic Overview of the Interplay of Traditional Cardiovascular Risk Factors and Genetic Variants Contributing to Disease Risk in Our Study Sample

In analysis of coronary events and ischemic strokes separately, *F5* SNP *rs7542281* showed an association with both coronary and ischemic stroke events as a single variant in women, and *F5 rs2269648* was associated with incident ischemic stroke. In men, *PROC rs1401296*, together with *F5* Leiden mutation, was associated with incident ischemic stroke. The wide confidence intervals seen when analyzing stroke events in females reflect the small number of female stroke cases. Several other SNPs showed a suggestive association with the endpoints, but failed the FDR <0.1 criterion (Tables 5 and 6). Figure 2 shows a schematic overview of the contribution of the gene variants analyzed here to CVD.

**Table 6:**
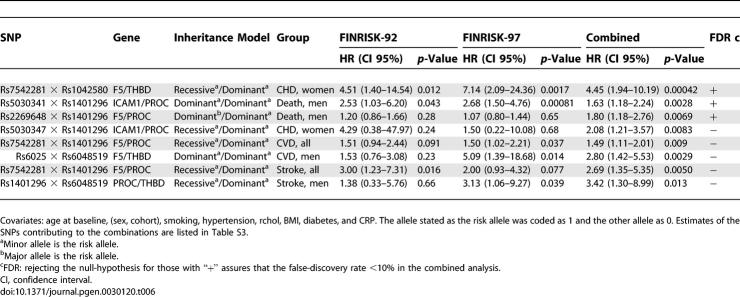
Association of Pair-Wise SNP Combinations with the Endpoints in Time-to-Event Analysis

The importance of the SNPs excluded from further analyses was assessed by analysis of all the 36 SNPs with Cox's proportional hazards model, assuming dominant inheritance, using combined cohorts in all the data and then with separate sexes. These results strongly suggest that the two-stage approach was able to recognize all informative SNPs: the significant results (*p* < 0.05) seen in Cox's model were concentrated in those SNPs selected for stage 2. With all the other SNPs showing statistical significance for the combined cohorts, the results from the separate study cohorts showed either a discrepancy in the hazard ratio or only one of the cohorts gave a signal (Tables S4–S16).

## Discussion

Our present study provides a fresh way to utilize different methods to analyze the impact of multiple genes on a phenotype. This two-step analysis strategy used classification-trees to select a subset of SNPs from a larger set of genome-wide haplotype tagging SNPs for further genetic analyses of allelic covariance and hazard ratios. The importance of the SNPs excluded from the further analyses was assessed with a separate sensitivity analysis. Even though all genetic variants, or combinations of them, were required to show consistent effects in both the independent cohorts to be considered significant, only functional studies and replications in other populations will validate the importance of these findings for CVD. This may prove a valuable approach in whole-genome association analyses, where the amount of genetic data is overwhelming.

Classification-trees structure data by searching first for the variable best explaining the determined endpoint and splitting the dataset into subgroups according to this variable. The search continues in the specified subgroups, which again split into smaller groups accordingly. Classification-trees are thus useful in organizing noisy datasets comprising multiple variables, and can be used to identify specific pathways determining an outcome or specific subgroups having similarities leading to this outcome. Gruenewald et al. [28] utilized classification-trees to identify risk markers for mortality in an elderly population, and determined several mortality-predictive pathways consisting of different combinations of markers, in both men and women. Their study highlights the multiplicity of solutions leading to the same outcome and illustrates how classification-trees can prove useful in structuring multiple variables in an organized manner. In our data, the classification-tree analysis itself failed to detect consistent gene–gene or gene–environment interaction patterns, but was useful in reducing the number of SNPs analyzed with other models. In our dataset, the forest of trees demonstrated that the influence of any tested SNP on cardiovascular events was evident only after first splitting a tree by one or more traditional risk factors, such as cholesterol level, BMI, or diabetes. This fits well the current understanding of the nature of the complex diseases: setting aside some rare, strictly familial cases of CVD, the vast majority of cardiovascular diseases are likely to be characterized by several low-predisposing genes and their possible interactions. In these cases, the traditional phenotypic risk factors play a stronger role, and genetic factors are more likely to lurk in the background. Classification-trees could therefore prove powerful also in determining subsets, “branches,” of individuals among whom the genetic variants are especially evident risk factors. For example, the *F5* Leiden mutation has been suggested to be associated with CVD among young women with an unhealthy lifestyle [29].

Our study identified several thrombosis-related risk variants—both single SNPs and their pair-wise combinations—for coronary events, ischemic strokes, and total mortality, showing consistent association in two independently selected Finnish population cohorts. Thrombosis is a central step in the pathogenesis of myocardial infarction and ischemic stroke. The ruptured wall of a coronary plaque is covered with thrombosis leading either to local obstruction of circulation or emboli traveling in more peripheral arterial branches. Thus, variants in thrombosis genes are also biologically relevant risk factors for coronary and stroke events. Our observations on various allelic combinations from several genes associating with CVD events highlight the importance of wide-perspective studies concentrating on numerous genes and their allelic variants to explore the genetic background of any complex trait. One should proceed cautiously when drawing biological conclusions from statistical interaction models: the underlying biological processes are likely to be highly complex, and evidence of combined effects in a statistical sense fails to do justice to this complexity. Our results indicate, however, that SNP combinations may reveal risk variants that could remain unnoticed when one is concentrating purely on single SNPs; SNP combinations could therefore be one step forward in the study of complex traits. Here, the CVD-associated variants of the candidate genes, excluding the Leiden mutation, represent either intronic or intragenic SNPs, likely linked to other, functional variants contributing to the true disease predisposition.

Studies on the *F5* Leiden mutation in arterial thrombosis have been controversial [30], but a recent meta-analysis suggests that the *F5* Leiden mutation contributes significantly to coronary events [31]. The *F5* Leiden mutation also associated with ischemic stroke in children [32]. In our study, men carrying the risk (minor) allele of *F5* Leiden were at a 4.47-fold increased risk for ischemic stroke, and a suggestive finding emerged when women were included in the analysis. The Leiden mutation also contributed to CVD risk in men jointly with a *THBD* SNP *rs6048519*. Addressing potential interactions for rare allelic variants, or to analyze the real impact of the Leiden mutation, requires very large datasets [31]. Our earlier results implied that in our study cohorts *THBD* variants alone seemed to play no role in CVD [15], but our new data from this study suggest that *THBD* may contribute to CVD in combination with other risk factors. Podgoreanu and colleagues reported an association of *ICAM1* SNP *rs5498* with postoperative myocardial infarction [19]. This SNP was in our population in strong LD (*r* ^2^ = 0.82) with SNP *rs3093030,* but with no association with CVD. The wide confidence intervals in the analyses of female stroke cases reflect the need for larger study cohorts. In the present study, the number of female stroke participants was especially limited.

This is, to our knowledge, the first study to cover all known common allelic variants in *F5, ICAM1, PROC,* and *THBD* genes, analyzing them as a physiological “pathway” entity and assessing their relationship with the traditional CVD risk factors. Multiple testing is an emerging issue in all complex genetics studies comprising several markers. We adjusted the findings of the final analysis step for multiple testing using the FDR, although this does not adjust for the fact that the earlier selection of SNPs by use of classification-trees was done on the same dataset. We chose to use 10% as a limit in the FDR analyses, stating that with this limit, 90% of the findings are expected to be true positives, whereas 10% could still be false positives. In addtition, the sensitivity analysis suggested that the classification-tree approach was able to select the informative SNPs, excluding the less informative ones. The major strength of this study is to further minimize the possibility of false-positive findings by requiring consistency in the findings in two separate cohorts of the same population. However, these results still need replication in other independent studies and populations. The major limitation of this study is its cohort size; studies comprising several hundred cases in each category are needed to profoundly address gene–gene interactions, especially once these types of analyses are reaching genome-wide datasets of thousands of genes. Another limitation of the study sample is the lack of plasma concentrations of the hemostatic factors.

In conclusion, combining data from several genes encoding components of the same biological pathway and analyzing the impact of genes together with other relevant risk factors may prove useful in regard to studying diseases with polygenic structures. Currently, sensible ways are called for to analyze the overwhelming information load produced by studies containing various genes and many variants. Our approach provides one example for selecting the informative variants from the background. Our results from the thrombosis cascade of four genes shed light on the interplay of these gene variants' roles in arterial thrombosis.

## Supporting Information

Figure S1 Linkage Disequilibrium Pattern (*r* ^2^) and Haplotype Blocks of the Coagulation Factor V SNPs Genotyped (71 KB JPG)

Table S1 Significant Splits (Sample-Specific *p*-Value, χ^2^) Seen in Classification-Trees Grown in AnswerTree3.0 with Ten Random Male Datasets, Each Containing 60% of the Original Data SNPs were analyzed as 0 (minor allele carriers) to 1 (major allele homozygotes).
(11 KB DOC)

Table S2 Minor Allele Frequencies of All SNPs Analyzed (141 KB DOC)

Table S3 Estimates for the SNPs Contributing to the Pair-Wise SNP Combinations Presented in Table S6
(12 KB DOC)

Table S4 Association of the SNPs Studied with Incident Coronary Events in Time-to-Event Analysis in Both Sexes Covariates: age at baseline, (sex, cohort), smoking, hypertension, TC/HDL, BMI, diabetes, and CRP). FINRISK-92 and FINRISK-97 cohorts combined for the analysis. Analysis performed according to dominant inheritance model; hazard ratios >1 show major allele as the risk allele.
(12 KB DOC)

Table S5 Association of the SNPs Studied with Incident Coronary Events in Time-to-Event Analysis in Men Covariates: age at baseline, (sex, cohort), smoking, hypertension, TC/HDL, BMI, diabetes, and CRP. FINRISK-92 and FINRISK-97 cohorts combined for the analysis. Analysis performed according to dominant inheritance model; hazard ratios >1 show major allele as the risk allele.
(12 KB DOC)

Table S6 Association of the SNPs Studied with Incident Coronary Events in Time-to-Event Analysis in Women Covariates: age at baseline, (sex, cohort), smoking, hypertension, TC/HDL, BMI, diabetes, and CRP. FINRISK-92 and FINRISK-97 cohorts combined for the analysis. Analysis performed according to dominant inheritance model; hazard ratios >1 show major allele as the risk allele.
(12 KB DOC)

Table S7 Association of the SNPs Studied with Incident Ischemic Stroke Events in Time-to-Event Analysis in Both Sexes Covariates: age at baseline, (sex, cohort), smoking, hypertension, TC/HDL, BMI, diabetes, and CRP. FINRISK-92 and FINRISK-97 cohorts combined for the analysis, which comprises both sexes. Analysis performed according to dominant inheritance model; hazard ratios >1 show major allele as the risk allele.
(12 KB DOC)

Table S8 Association of the SNPs Studied with Incident Ischemic Stroke Events in Time-to-Event Analysis in Men Covariates: age at baseline, (sex, cohort), smoking, hypertension, TC/HDL, BMI, diabetes, and CRP. FINRISK-92 and FINRISK-97 cohorts combined for the analysis. Analysis performed according to dominant inheritance model; hazard ratios >1 show major allele as the risk allele.
(12 KB DOC)

Table S9 Association of the SNPs Studied with Incident Ischemic Stroke Events in Time-to-Event Analysis in Women Covariates: age at baseline, (sex, cohort), smoking, hypertension, TC/HDL, BMI, diabetes, and CRP. FINRISK-92 and FINRISK-97 cohorts combined for the analysis. Analysis performed according to dominant inheritance model; hazard ratios >1 show major allele as the risk allele.
(12 KB DOC)

Table S10 Association of the SNPs Studied with Incident Cardiovascular (Coronary or Ischemic stroke) Events in Time-to-Event Analysis in Both Sexes Covariates: age at baseline, (sex, cohort), smoking, hypertension, TC/HDL, BMI, diabetes, and CRP. FINRISK-92 and FINRISK-97 cohorts combined for the analysis. Analysis performed according to dominant inheritance model; hazard ratios >1 show major allele as the risk allele.
(12 KB DOC)

Table S11 Association of the SNPs Studied with Incident Cardiovascular (Coronary or Ischemic Stroke) Events in Time-to-Event Analysis in Men Covariates: age at baseline, (sex, cohort), smoking, hypertension, TC/HDL, BMI, diabetes, and CRP. FINRISK-92 and FINRISK-97 cohorts combined for the analysis. Analysis performed according to dominant inheritance model; hazard ratios >1 show major allele as the risk allele.
(12 KB DOC)

Table S12 Association of the SNPs Studied with Incident Cardiovascular (Coronary or Ischemic Stroke) Events in Time-to-Event Analysis in Women Covariates: age at baseline, (sex, cohort), smoking, hypertension, TC/HDL, BMI, diabetes, and CRP. FINRISK-92 and FINRISK-97 cohorts combined for the analysis. Analysis performed according to dominant inheritance model; hazard ratios >1 show major allele as the risk allele.
(12 KB DOC)

Table S13 Association of the SNPs Studied with Total Mortality in Time-to-Event Analysis in Both Sexes Covariates: age at baseline, (sex, cohort), smoking, hypertension, TC/HDL, BMI, diabetes, and CRP). FINRISK-92 and FINRISK-97 cohorts combined for the analysis. Analysis performed according to dominant inheritance model; hazard ratios >1 show major allele as the risk allele.
(12 KB DOC)

Table S14 Association of the SNPs Studied with Incident Cardiovascular (Coronary or Ischemic Stroke) Events in Time-to-Event Analysis in Men Covariates: age at baseline, (sex, cohort), smoking, hypertension, TC/HDL, BMI, diabetes, and CRP. FINRISK-92 and FINRISK-97 cohorts combined for the analysis. Analysis performed according to dominant inheritance model; hazard ratios >1 show major allele as the risk allele.
(12 KB DOC)

Table S15 Association of the SNPs Studied with Total Mortality in Time-to-Event Analysis in Women Covariates: age at baseline, (sex, cohort), smoking, hypertension, TC/HDL, BMI, diabetes, and CRP). FINRISK-92 and FINRISK-97 cohorts combined for the analysis. Analysis performed according to dominant inheritance model; hazard ratios >1 show major allele as the risk allele.
(12 KB DOC)

Table S16 SNPs Not Chosen for Stage 2 Analyses Based on the Classification-Trees and Showing *p*-Values <0.05 in the Analysis for Combined Study Cohorts Analyzed in the Separate Cohorts FINRISK-92 and FINRISK-97 (12 KB DOC)

### Accession Numbers

The National Center for Biotechnology Information LocusLink (http://www.ncbi.nlm.nih.gov/sites/entrez) GeneID numbers for the genes discussed in this paper are Coagulation factor V (F5), 2153; Intercellular adhesion molecule 1, 3383; Protein C, 5624; and Thrombomodulin, 7056.

## Abbreviations

BMI: body-mass index
CHD: coronary heart disease
CI: confidence interval
CRP: C-reactive protein
CVD: cardiovascular diseases
FDR: false-discovery rate
F5: coagulation factor V
HDL: high-density lipoprotein
HR: hazard ratio
ICAM1: intercellular adhesion molecule 1
ICD: International Classification of Diseases
LD: linkage disequilibrium
non-HDL: total cholesterol−high-density cholesterol
PROC: protein C
SD: standard deviation
SNP: single nucleotide polymorphism
TC: total cholesterol
TC/HDL: total cholesterol to high-density cholesterol ratio
TG: triglycerides
THBD: thrombomodulin
WHR: waist-to-hip ratio
