# Supplementary figures and images for: Combined Effects of Thrombosis Pathway Gene Variants Predict Cardiovascular Events
Source: PLoS Genet. 2007 Jul 27;3(7):e120. doi: 10.1371/journal.pgen.0030120 (PMC1934395; doi:10.1371/journal.pgen.0030120)

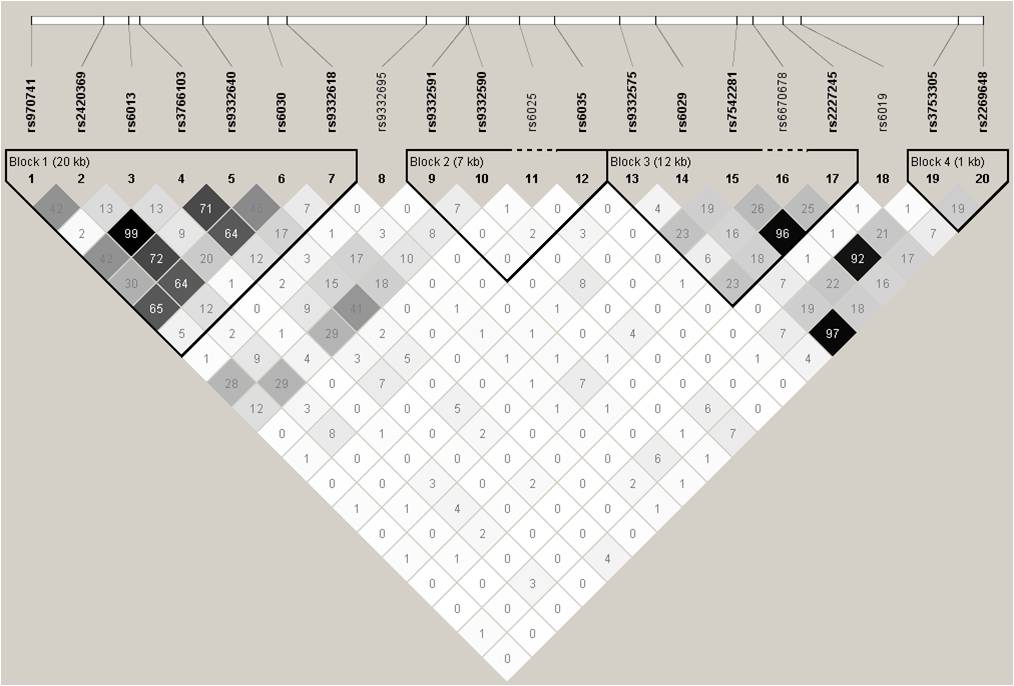

Supplement: Figure S1 — (71 KB JPG) [file pgen.0030120.sg001.jpg]
