# Supplementary material for: Combined Effects of Thrombosis Pathway Gene Variants Predict Cardiovascular Events
Source: PLoS Genet. 2007 Jul 27;3(7):e120. doi: 10.1371/journal.pgen.0030120 (PMC1934395; doi:10.1371/journal.pgen.0030120)
Supplement: Table S1 — SNPs were analyzed as 0 (minor allele carriers) to 1 (major allele homozygotes). (11 KB DOC) [file pgen.0030120.st001.doc]

Supplementary Table 1: Significant splits (sample specific p-value, chi-square) seen in classification trees grown in AnswerTree3.0 with 10 random male datasets, each containing 60% of the original data. SNPs were analyzed as 0 (minor allele carriers) to 1 (major allele homozygotes).

| Tree N.o. | 1 | 2 | 3 | 4 | 5 | 6 | 7 | 8 | 9 | 10 |
| --- | --- | --- | --- | --- | --- | --- | --- | --- | --- | --- |
| Diabetes | - | 0.00080 | 0.000029 | 0.000053 | 0.0012 | 0.00035 | - | - | 0.00043 | 0.0000075 |
| BMI ^a^ | 0.00021 | 0.000076 | 0.000014 | 0.0013 | 0.000071 | 0.00024 | 0.00069 | 0.0018 | 0.0010 | 0.000036 |
| HDL ^b^ | - | - | 0.000076 | - | - | - | - | - | 0.0016 | - |
| Non-HDL ^c^ | - | 0.00064 | - | - | 0.00037 | 0.00085 | 0.0041 | - | - | - |
| TC/HDL ^d^ | 0.00021 | 0.0011 | 0.000052 | 0.00090 | 0.0017 | 0.0000025 | 0.0028 | 0.0012 | 0.000019 | - |
| CRP ^e^ | 0.00015 | - | - | 0.00082 | 0.000037 | 0.0010 | 0.0014 | 0.00038 | - | 0.00033 |
| Hypertension | 0.00047 | - | 0.00099 | - | - | - | 0.0037 | 0.00060 | - | 0.00016 |
| TG ^f^ | 0.00036 | - | - | - | - | - | - | 0.0022 | 0.00047 | - |
| WHR ^g^ | - | - | - | 0.00032 | - | - | - | - | - | 0.0000099 |
| *Rs3753305* | - | 0.00054 | - | - | - | - | - | - | - | - |

^a^ BMI: Body-mass index ^b^ HDL: High-density lipoprotein cholesterol ^c^ non-HDL: Total cholesterol – HDL ^d^ CRP: C-reactive protein ^e^ TG: Triglycerides ^f^ WHR: Waist-to-hip ratio
