# Supplementary material for: Combined Effects of Thrombosis Pathway Gene Variants Predict Cardiovascular Events
Source: PLoS Genet. 2007 Jul 27;3(7):e120. doi: 10.1371/journal.pgen.0030120 (PMC1934395; doi:10.1371/journal.pgen.0030120)
Supplement: Table S2 — (141 KB DOC) [file pgen.0030120.st002.doc]

Supplementary Table 2: Minor allele frequencies of all SNPs analyzed.

| SNP | Gene | Minor  Allele | FINRISK-92  Men  CVD ^a^ | Men  Subcohort ^b^ | Women  CVD ^a^ | Women  Subcohort ^b^ | FINRISK-97  Men  CVD ^a^ | Men  Subcohort ^b^ | Women  CVD ^a^ | Women  Subcohort ^b^ |
| --- | --- | --- | --- | --- | --- | --- | --- | --- | --- | --- |
| *Rs970741* | *F5* | *G* | 0.19 | 0.19 | 0.13 | 0.23 | 0.20 | 0.20 | 0.20 | 0.21 |
| *Rs2420369* | *F5* | *T* | 0.37 | 0.38 | 0.31 | 0.42 | 0.41 | 0.36 | 0.36 | 0.42 |
| *Rs6013* | *F5* | *T* | 0.08 | 0.08 | 0.09 | 0.09 | 0.07 | 0.09 | 0.08 | 0.09 |
| *Rs9332640* | *F5* | *G* | 0.43 | 0.46 | 0.45 | 0.47 | 0.47 | 0.45 | 0.42 | 0.49 |
| *Rs6030* | *F5* | *G* | 0.28 | 0.27 | 0.23 | 0.32 | 0.28 | 0.29 | 0.28 | 0.31 |
| *Rs9332618* | *F5* | *A* | 0.17 | 0.16 | 0.18 | 0.19 | 0.16 | 0.16 | 0.22 | 0.18 |
| *Rs9332695* | *F5* | *T* | 0.05 | 0.06 | 0.05 | 0.02 | 0.05 | 0.03 | 0.04 | 0.02 |
| *Rs9332591* | *F5* | *A* | 0.12 | 0.13 | 0.11 | 0.13 | 0.10 | 0.12 | 0.13 | 0.17 |
| *Rs9332590* | *F5* | *G* | 0.34 | 0.34 | 0.33 | 0.29 | 0.34 | 0.34 | 0.32 | 0.26 |
| *Rs6025* | *F5* | *T* | 0.03 | 0.03 | 0.03 | 0.01 | 0.03 | 0.01 | 0.03 | 0.03 |
| *Rs6035* | *F5* | *C* | 0.03 | 0.03 | 0.04 | 0.07 | 0.06 | 0.05 | 0.04 | 0.05 |
| *Rs9332575* | *F5* | *C* | 0.14 | 0.10 | 0.11 | 0.08 | 0.10 | 0.12 | 0.15 | 0.11 |
| *Rs7542281* | *F5* | *T* | 0.37 | 0.34 | 0.36 | 0.26 | 0.32 | 0.35 | 0.43 | 0.33 |
| *Rs6019* | *F5* | *C* | 0.02 | 0.02 | 0.02 | 0.04 | 0.03 | 0.03 | 0.04 | 0.02 |
| *Rs3753305* | *F5* | *C* | 0.37 | 0.38 | 0.33 | 0.36 | 0.36 | 0.37 | 0.32 | 0.32 |
| *Rs2269648* | *F5* | *T* | 0.24 | 0.28 | 0.23 | 0.30 | 0.27 | 0.25 | 0.24 | 0.29 |
| *Rs5030390* | *ICAM1* | *A* | 0.02 | 0.04 | 0.05 | 0.05 | 0.04 | 0.04 | 0.01 | 0.04 |
| *Rs5030347* | *ICAM1* | *A* | 0.23 | 0.21 | 0.23 | 0.16 | 0.23 | 0.18 | 0.24 | 0.17 |
| *Rs281432* | *ICAM1* | *G* | 0.45 | 0.42 | 0.48 | 0.48 | 0.44 | 0.46 | 0.42 | 0.49 |
| *Rs3093032* | *ICAM1* | *T* | 0.13 | 0.13 | 0.16 | 0.19 | 0.13 | 0.14 | 0.15 | 0.14 |
| *Rs3093030* | *ICAM1* | *T* | 0.41 | 0.41 | 0.39 | 0.40 | 0.42 | 0.43 | 0.43 | 0.36 |
| *Rs5030341* | *ICAM1* | del*CT* ^c^ | 0.33 | 0.33 | 0.37 | 0.38 | 0.30 | 0.34 | 0.30 | 0.37 |
| *Rs1799810* | *PROC* | *T* | 0.33 | 0.35 | 0.39 | 0.33 | 0.35 | 0.33 | 0.25 | 0.35 |
| *Rs2069920* | *PROC* | *C* | 0.43 | 0.42 | 0.43 | 0.46 | 0.42 | 0.42 | 0.43 | 0.39 |
| *Rs2069923* | *PROC* | *C* | 0.03 | 0.03 | 0.04 | 0.03 | 0.02 | 0.03 | 0.01 | 0.04 |
| *Rs2069928* | *PROC* | *T* | 0.24 | 0.21 | 0.18 | 0.21 | 0.24 | 0.24 | 0.29 | 0.25 |
| *Rs5937* | *PROC* | *A* | 0.27 | 0.31 | 0.31 | 0.34 | 0.37 | 0.35 | 0.38 | 0.39 |
| *Rs1401296* | *PROC* | *C* | 0.31 | 0.28 | 0.33 | 0.28 | 0.36 | 0.36 | 0.30 | 0.31 |
| *Rs6113909* | *THBD* | *G* | 0.37 | 0.38 | 0.41 | 0.37 | 0.44 | 0.45 | 0.39 | 0.39 |
| *Rs6082986* | *THBD* | *A* | 0.31 | 0.28 | 0.33 | 0.28 | 0.36 | 0.36 | 0.30 | 0.31 |
| *Rs1962* | *THBD* | *C* | 0.15 | 0.17 | 0.14 | 0.20 | 0.16 | 0.17 | 0.17 | 0.14 |
| *Rs3176123* | *THBD* | *G* | 0.32 | 0.33 | 0.33 | 0.32 | 0.29 | 0.25 | 0.30 | 0.31 |
| *Rs1042580* | *THBD* | *C* | 0.30 | 0.30 | 0.32 | 0.29 | 0.37 | 0.36 | 0.33 | 0.31 |
| *Rs3176119* | *THBD* | *C* | 0.06 | 0.03 | 0.02 | 0.03 | 0.03 | 0.03 | 0.04 | 0.04 |
| *Rs3216183* | *THBD* | ins*TT* ^d^ | 0.19 | 0.21 | 0.18 | 0.21 | 0.16 | 0.13 | 0.18 | 0.17 |
| *Rs6048519* | *THBD* | *T* | 0.43 | 0.43 | 0.44 | 0.46 | 0.43 | 0.48 | 0.45 | 0.37 |

^a^ CVD: Incident coronary or ischemic stroke event during follow-up

^b^ Individuals with any CVD events removed from subcohorts.

^c^ Del: Deletion ^d^ Ins: Insertion
