# Supplementary material for: Combined Effects of Thrombosis Pathway Gene Variants Predict Cardiovascular Events
Source: PLoS Genet. 2007 Jul 27;3(7):e120. doi: 10.1371/journal.pgen.0030120 (PMC1934395; doi:10.1371/journal.pgen.0030120)
Supplement: Table S3 — (12 KB DOC) [file pgen.0030120.st003.doc]

Supplementary Table 3: Estimates for the SNPs contributing to the pair-wise SNP combinations presented in Table 6.

| SNP | Gene | Inheritance  Model | Group | FINRISK-92  HR (CI 95%) p | FINRISK-97  HR (CI 95%) p | Combined  HR (CI 95%) p |
| --- | --- | --- | --- | --- | --- | --- |
| *Rs7542281* | *F5* | Recessive ^a^ | CHD women | 2.28 (0.84-6.19) 0.11 | 3.65 (1.62-8.21) 0.0017 | 2.52 (1.39-4.55) 0.0022 |
| *Rs1042580* | *THBD* | Dominant ^a^ | CHD women | 1.95 (0.93-4.10) 0.078 | 0.95 (0.47-1.91) 0.89 | 1.19 (0.72-1.95) 0.50 |
| *Rs5030341* | *ICAM1* | Dominant ^a^ | Death men | 1.17 (0.82-1.66) 0.41 | 1.11 (0.83-1.48) 0.47 | 1.13 (0.91-1.41) 0.29 |
| *Rs1401296* | *PROC* | Dominant ^a^ | Death men | 1.02 (0.71-1.45) 0.92 | 1.03 (0.78-1.37) 0.82 | 1.04 (0.83-1.29) 0.74 |
| *Rs2269648* | *F5* | Dominant ^b^ | Death men | 1.40 (0.78-2.50) 0.26 | 0.99 (0.75-1.30) 0.93 | 0.87 (0.61-1.24) 0.45 |
| *Rs5030347* | *ICAM1* | Recessive ^a^ | CHD women | 4.81 (1.68-13.77) 0.0034 | 1.25 (0.31-5.01) 0.75 | 1.13 (0.70-1.81) 0.62 |
| *Rs1401296* | *PROC* | Dominant ^a^ | CHD women | 1.65 (0.82-3.30) 0.16 | 1.30 (0.68-2.49) 0.43 | 1.44 (0.90-2.31) 0.14 |
| *Rs7542281* | *F5* | Recessive ^a^ | CVD all | 1.24 (0.81-1.89) 0.32 | 1.24 (0.86-1.78) 0.24 | 1.22 (0.93-1.60) 0.15 |
| *Rs1401296* | *PROC* | Dominant ^a^ | CVD all | 1.03 (0.76-1.41) 0.83 | 1.11 (0.83-1.50) 0.49 | 1.07 (0.89-1.29) 0.46 |
| *Rs6025* | *F5* | Dominant ^a^ | CVD men | Very rare ^c^ | Very rare ^c^ | 1.55 (0.92-2.61) 0.11 |
| *Rs6048519* | *THBD* | Dominant ^a^ | CVD men | 1.10 (0.73-1.65) 0.64 | 1.02 (0.73-1.43) 0.91 | 1.02 (0.78-1.32) 0.90 |
| *Rs7542281* | *F5* | Recessive ^a^ | Stroke all | 2.71 (1.24-5.95) 0.013 | 1.25 (0.61-2.58) 0.55 | 2.07 (1.17-3.65) 0.012 |
| *Rs1401296* | *PROC* | Dominant ^a^ | Stroke all | 0.96 (0.58-1.57) 0.86 | 1.27 (0.76-2.13) 0.37 | 1.22 (0.79-1.89) 0.36 |
| *Rs1401296* | *PROC* | Recessive ^a^ | Stroke men | 3.16 (1.11-9.00) 0.031 | 2.33 (1.12-4.88) 0.025 | 2.84 (1.32-6.12) 0.0078 |
| *Rs6048519* | *THBD* | Dominant ^a^ | Stroke men | 2.09 (0.71-6.18) 0.18 | 0.93 (0.48-1.81) 0.83 | 1.07 (0.56-2.04) 0.83 |

^a^ The minor allele is the risk allele, ^b^ The major allele is the risk allele, ^c^ Could not be analyzed in separate cohorts (number of cases <10).
