# Supplementary material for: Combined Effects of Thrombosis Pathway Gene Variants Predict Cardiovascular Events
Source: PLoS Genet. 2007 Jul 27;3(7):e120. doi: 10.1371/journal.pgen.0030120 (PMC1934395; doi:10.1371/journal.pgen.0030120)
Supplement: Table S4 — Covariates: age at baseline, (sex, cohort), smoking, hypertension, TC/HDL, BMI, diabetes, and CRP). FINRISK-92 and FINRISK-97 cohorts combined for the analysis. Analysis performed according to dominant inheritance model; hazard ratios >1 show major allele as the risk allele. (12 KB DOC) [file pgen.0030120.st004.doc]

Supplementary Table 4: Association of the SNPs studied with incident coronary events in time-to-event analysis (covariates: age at baseline, (sex, cohort), smoking, hypertension, TC/HDL, BMI, diabetes, CRP). FINRISK-92 and FINRISK-97 cohorts combined for the analysis, which comprises both sexes. Analysis performed according to dominant inheritance model; hazard ratios >1 show major allele as the risk allele.

| SNP | Gene | Hazard Ratio | 95% Confidence  Interval | p-value |
| --- | --- | --- | --- | --- |
| ***Rs2420369*** | ***F5*** | **1.17** | **9.93-1.48** | **0.1733** |
| ***Rs9332591*** | ***F5*** | **1.14** | **0.87-1.50** | **0.3408** |
| ***Rs6025*** | ***F5*** | **1.22** | **0.74-2.02** | **0.4414** |
| ***Rs7542281*** | ***F5*** | **1.11** | **0.80-1.54** | **0.5404** |
| ***Rs2269648*** | ***F5*** | **1.11** | **0.89-1.40** | **0.3488** |
| ***Rs5030347*** | ***ICAM1*** | **0.97** | **0.95-1.06** | **0.0546** |
| ***Rs5030341*** | ***ICAM1*** | **1.25** | **0.98-1.58** | **0.0678** |
| ***Rs5937*** | ***PROC*** | **1.21** | **0.97-1.51** | **0.1001** |
| ***Rs1401296*** | ***PROC*** | **1.04** | **0.83-1.31** | **0.7216** |
| ***Rs1042580*** | ***THBD*** | **0.96** | **0.76-1.21** | **0.7252** |
| ***Rs6048519*** | ***THBD*** | **0.98** | **0.77-1.24** | **0.8465** |
| *Rs970741* | *F5* | 1.04 | 0.82-1.32 | 0.7543 |
| *Rs6013* | *F5* | 1.12 | 0.82-1.54 | 0.4846 |
| *Rs9332640* | *F5* | 1.22 | 0.96-1.54 | 0.1097 |
| *Rs6030* | *F5* | 1.11 | 0.89-1.39 | 0.3467 |
| *Rs9332618* | *F5* | 0.93 | 0.73-1.81 | 0.5568 |
| *Rs9332695* | *F5* | 0.85 | 0.57-1.27 | 0.4334 |
| *Rs9332590* | *F5* | 1.03 | 0.82-1.29 | 0.8007 |
| *Rs6035* | *F5* | 1.26 | 0.87-1.81 | 0.2248 |
| *Rs9332575* | *F5* | 1.05 | 0.79-1.40 | 0.7370 |
| *Rs6019* | *F5* | 1.13 | 0.70-1.81 | 0.6275 |
| *Rs3753305* | *F5* | 0.98 | 0.78-1.24 | 0.8692 |
| *Rs5030390* | *ICAM1* | 1.23 | 0.79-1.91 | 0.3689 |
| *Rs281432* | *ICAM1* | 1.22 | 0.96-1.55 | 0.1113 |
| *Rs3093032* | *ICAM1* | 1.08 | 0.83-1.40 | 0.5697 |
| *Rs3093030* | *ICAM1* | 1.04 | 0.82-1.31 | 0.7657 |
| *Rs1799810* | *PROC* | 1.13 | 0.91-1.41 | 0.2684 |
| *Rs2069920* | *PROC* | 0.79 | 0.62-1.01 | 0.0619 |
| *Rs2069923* | *PROC* | 1.11 | 0.69-1.80 | 0.6617 |
| *Rs2069928* | *PROC* | 0.96 | 0.76-1.21 | 0.7186 |
| *Rs6113909* | *THBD* | 0.98 | 0.78-1.24 | 0.8873 |
| *Rs6082986* | *THBD* | 1.00 | 0.80-1.26 | 0.9755 |
| *Rs1962* | *THBD* | 1.03 | 0.81-1.32 | 0.7869 |
| *Rs3176123* | *THBD* | 0.94 | 0.75-1.17 | 0.5658 |
| *Rs3176119* | *THBD* | 0.79 | 0.52-1.22 | 0.2914 |
| *Rs3216183* | *THBD* | 1.02 | 0.80-1.31 | 0.8519 |
