# Supplementary material for: Combined Effects of Thrombosis Pathway Gene Variants Predict Cardiovascular Events
Source: PLoS Genet. 2007 Jul 27;3(7):e120. doi: 10.1371/journal.pgen.0030120 (PMC1934395; doi:10.1371/journal.pgen.0030120)
Supplement: Table S5 — Covariates: age at baseline, (sex, cohort), smoking, hypertension, TC/HDL, BMI, diabetes, and CRP. FINRISK-92 and FINRISK-97 cohorts combined for the analysis. Analysis performed according to dominant inheritance model; hazard ratios >1 show major allele as the risk allele. (12 KB DOC) [file pgen.0030120.st005.doc]

Supplementary Table 5: Association of the SNPs studied with incident coronary events in time-to-event analysis (covariates: age at baseline, (sex, cohort), smoking, hypertension, TC/HDL, BMI, diabetes, CRP) in men. FINRISK-92 and FINRISK-97 cohorts combined for the analysis. Analysis performed according to dominant inheritance model; hazard ratios >1 show major allele as the risk allele.

| SNP | Gene | Hazard Ratio | 95% Confidence  Interval | p-value |
| --- | --- | --- | --- | --- |
| ***Rs2420369*** | ***F5*** | **1.09** | **0.83-1.43** | **0.5510** |
| ***Rs9332591*** | ***F5*** | **1.23** | **0.88-1.72** | **0.2342** |
| ***Rs6025*** | ***F5*** | **1.40** | **0.76-2.57** | **0.2762** |
| ***Rs7542281*** | ***F5*** | **0.81** | **0.53-1.23** | **0.3118** |
| ***Rs2269648*** | ***F5*** | **1.03** | **0.79-1.34** | **0.8352** |
| ***Rs5030347*** | ***ICAM1*** | **0.98** | **0.96-1.01** | **0.2055** |
| ***Rs5030341*** | ***ICAM1*** | **1.25** | **0.95-1.64** | **0.1194** |
| ***Rs5937*** | ***PROC*** | **1.26** | **0.96-1.64** | **0.0933** |
| ***Rs1401296*** | ***PROC*** | **0.94** | **0.72-1.22** | **0.6348** |
| ***Rs1042580*** | ***THBD*** | **0.98** | **0.75-1.29** | **0.8800** |
| ***Rs6048519*** | ***THBD*** | **1.11** | **0.83-1.47** | **0.4837** |
| *Rs970741* | *F5* | 0.98 | 0.75-1.30 | 0.9114 |
| *Rs6013* | *F5* | 1.09 | 0.75-1.58 | 0.6602 |
| *Rs9332640* | *F5* | 1.25 | 0.94-1.65 | 0.1199 |
| *Rs6030* | *F5* | 1.10 | 0.84-1.44 | 0.4842 |
| *Rs9332618* | *F5* | 0.93 | 0.70-1.24 | 0.6293 |
| *Rs9332695* | *F5* | 0.72 | 0.46-1.15 | 0.1722 |
| *Rs9332590* | *F5* | 1.01 | 0.77-1.31 | 0.9703 |
| *Rs6035* | *F5* | 1.35 | 0.88-2.08 | 0.1726 |
| *Rs9332575* | *F5* | 1.27 | 0.89-1.81 | 0.1947 |
| *Rs6019* | *F5* | 1.17 | 0.65-2.09 | 0.6026 |
| *Rs3753305* | *F5* | 0.91 | 0.69-1.20 | 0.4891 |
| *Rs5030390* | *ICAM1* | 1.10 | 0.65-1.88 | 0.7246 |
| *Rs281432* | *ICAM1* | 1.27 | 0.96-1.69 | 0.0965 |
| *Rs3093032* | *ICAM1* | 1.23 | 0.89-1.68 | 0.2073 |
| *Rs3093030* | *ICAM1* | 0.98 | 0.74-1.30 | 0.8890 |
| *Rs1799810* | *PROC* | 1.14 | 0.88-1.49 | 0.3214 |
| *Rs2069920* | *PROC* | 0.87 | 0.65-1.16 | 0.3418 |
| *Rs2069923* | *PROC* | 1.02 | 0.60-1.75 | 0.9361 |
| *Rs2069928* | *PROC* | 1.05 | 0.80-1.38 | 0.7071 |
| *Rs6113909* | *THBD* | 1.02 | 0.77-1.35 | 0.8875 |
| *Rs6082986* | *THBD* | 1.02 | 0.78-1.34 | 0.8903 |
| *Rs1962* | *THBD* | 1.02 | 0.77-1.35 | 0.8792 |
| *Rs3176123* | *THBD* | 0.93 | 0.71-1.21 | 0.5860 |
| *Rs3176119* | *THBD* | 0.74 | 0.46-1.20 | 0.2255 |
| *Rs3216183* | *THBD* | 1.00 | 0.75-1.34 | 0.9779 |
