# Supplementary material for: Combined Effects of Thrombosis Pathway Gene Variants Predict Cardiovascular Events
Source: PLoS Genet. 2007 Jul 27;3(7):e120. doi: 10.1371/journal.pgen.0030120 (PMC1934395; doi:10.1371/journal.pgen.0030120)
Supplement: Table S6 — Covariates: age at baseline, (sex, cohort), smoking, hypertension, TC/HDL, BMI, diabetes, and CRP. FINRISK-92 and FINRISK-97 cohorts combined for the analysis. Analysis performed according to dominant inheritance model; hazard ratios >1 show major allele as the risk allele. (12 KB DOC) [file pgen.0030120.st006.doc]

Supplementary Table 6: Association of the SNPs studied with incident coronary events in time-to-event analysis (covariates: age at baseline, (sex, cohort), smoking, hypertension, TC/HDL, BMI, diabetes, CRP) in women. FINRISK-92 and FINRISK-97 cohorts combined for the analysis. Analysis performed according to dominant inheritance model; hazard ratios >1 show major allele as the risk allele.

| SNP | Gene | Hazard Ratio | 95% Confidence  Interval | p-value |
| --- | --- | --- | --- | --- |
| Rs2420369 | ***F5*** | **1.50** | **0.94-2.40** | **0.0877** |
| ***Rs9332591*** | ***F5*** | **1.05** | **0.61-1.79** | **0.8696** |
| ***Rs6025*** | F5 | **0.92** | **0.35-2.40** | **0.8635** |
| ***Rs7542281*** | ***F5*** | **2.52** | **1.39-4.55** | **0.0022** |
| ***Rs2269648*** | ***F5*** | **1.50** | **0.91-2.45** | **0.1097** |
| ***Rs5030347*** | ***ICAM1*** | **0.95** | **0.90-0.99** | **0.0525** |
| ***Rs5030341*** | ***ICAM1*** | **1.36** | **0.83-2.25** | **0.2242** |
| ***Rs5937*** | ***PROC*** | **1.05** | **0.67-1.63** | **0.8421** |
| ***Rs1401296*** | ***PROC*** | **1.44** | **0.90-2.31** | **0.1326** |
| ***Rs1042580*** | ***THBD*** | **0.84** | **0.51-1.38** | **0.4983** |
| ***Rs6048519*** | ***THBD*** | **0.68** | **0.41-1.15** | **0.1488** |
| *Rs970741* | *F5* | 1.32 | 0.80-2.18 | 0.2794 |
| *Rs6013* | *F5* | 1.33 | 0.71-2.53 | 0.3798 |
| *Rs9332640* | *F5* | 1.23 | 0.74-2.04 | 0.4190 |
| *Rs6030* | *F5* | 1.28 | 0.81-2.01 | 0.2902 |
| *Rs9332618* | *F5* | 1.14 | 0.69-1.88 | 0.6108 |
| *Rs9332695* | *F5* | 1.28 | 0.50-3.26 | 0.6076 |
| *Rs9332590* | *F5* | 0.94 | 0.59-1.50 | 0.7962 |
| *Rs6035* | *F5* | 0.92 | 0.44-1.95 | 0.8349 |
| *Rs9332575* | *F5* | 0.67 | 0.40-1.13 | 0.1338 |
| *Rs6019* | *F5* | 1.04 | 0.41-2.60 | 0.9396 |
| *Rs3753305* | *F5* | 1.13 | 0.71-1.78 | 0.6082 |
| *Rs5030390* | *ICAM1* | 1.93 | 0.71-5.27 | 0.1967 |
| *Rs281432* | *ICAM1* | 1.21 | 0.70-2.08 | 0.4898 |
| *Rs3093032* | *ICAM1* | 0.76 | 0.46-1.25 | 0.2753 |
| *Rs3093030* | *ICAM1* | 1.03 | 0.64-1.67 | 0.9025 |
| *Rs1799810* | *PROC* | 1.06 | 0.68-1.66 | 0.8072 |
| *Rs2069920* | *PROC* | 0.57 | 0.32-1.03 | 0.0626 |
| *Rs2069923* | *PROC* | 1.33 | 0.48-3.67 | 0.5800 |
| *Rs2069928* | *PROC* | 0.77 | 0.48-1.22 | 0.2603 |
| *Rs6113909* | *THBD* | 0.83 | 0.51-1.37 | 0.4736 |
| *Rs6082986* | *THBD* | 0.92 | 0.57-1.47 | 0.7243 |
| *Rs1962* | *THBD* | 1.14 | 0.68-1.90 | 0.6296 |
| *Rs3176123* | *THBD* | 0.94 | 0.60-1.48 | 0.7873 |
| *Rs3176119* | *THBD* | 0.76 | 0.29-2.09 | 0.5893 |
| *Rs3216183* | *THBD* | 1.02 | 0.62-1.67 | 0.9503 |
