# Supplementary material for: Combined Effects of Thrombosis Pathway Gene Variants Predict Cardiovascular Events
Source: PLoS Genet. 2007 Jul 27;3(7):e120. doi: 10.1371/journal.pgen.0030120 (PMC1934395; doi:10.1371/journal.pgen.0030120)
Supplement: Table S7 — Covariates: age at baseline, (sex, cohort), smoking, hypertension, TC/HDL, BMI, diabetes, and CRP. FINRISK-92 and FINRISK-97 cohorts combined for the analysis, which comprises both sexes. Analysis performed according to dominant inheritance model; hazard ratios >1 show major allele as the risk allele. (12 KB DOC) [file pgen.0030120.st007.doc]

Supplementary Table 7: Association of the SNPs studied with incident ischemic stroke events in time-to-event analysis (covariates: age at baseline, (sex, cohort), smoking, hypertension, TC/HDL, BMI, diabetes, CRP). FINRISK-92 and FINRISK-97 cohorts combined for the analysis, which comprises both sexes. Analysis performed according to dominant inheritance model; hazard ratios >1 show major allele as the risk allele.

| SNP | Gene | Hazard Ratio | 95% Confidence  Interval | p-value |
| --- | --- | --- | --- | --- |
| ***Rs2420369*** | ***F5*** | **1.26** | **0.75-2.12** | **0.3863** |
| ***Rs9332591*** | ***F5*** | **1.39** | **0.76-2.53** | **0.2885** |
| ***Rs6025*** | ***F5*** | **2.98** | **1.20-7.41** | **0.0191** |
| ***Rs7542281*** | ***F5*** | **2.46** | **1.00-4.42** | **0.0570** |
| ***Rs2269648*** | ***F5*** | **0.86** | **0.53-1.42** | **0.5629** |
| ***Rs5030347*** | ***ICAM1*** | **0.97** | **0.92-1.02** | **0.1838** |
| ***Rs5030341*** | ***ICAM1*** | **1.52** | **0.91-2.54** | **0.1068** |
| ***Rs5937*** | ***PROC*** | **1.04** | **0.67-1.62** | **0.8621** |
| ***Rs1401296*** | ***PROC*** | **1.48** | **0.87-2.52** | **0.1506** |
| ***Rs1042580*** | ***THBD*** | **0.98** | **0.59-1.64** | **0.9481** |
| ***Rs6048519*** | ***THBD*** | **1.28** | **0.79-2.09** | **0.3161** |
| *Rs970741* | *F5* | 1.19 | 0.72-1.96 | 0.4898 |
| *Rs6013* | *F5* | 1.41 | 0.72-2.76 | 0.3149 |
| *Rs9332640* | *F5* | 1.40 | 0.80-2.44 | 0.2350 |
| *Rs6030* | *F5* | 1.41 | 0.84-2.36 | 0.1854 |
| *Rs9332618* | *F5* | 1.02 | 0.60-1.71 | 0.9516 |
| *Rs9332695* | *F5* | 0.71 | 0.31-1.58 | 0.3960 |
| *Rs9332590* | *F5* | 1.10 | 0.71-1.70 | 0.6837 |
| *Rs6035* | *F5* | 2.19 | 1.02-4.72 | 0.0456 |
| *Rs9332575* | *F5* | 0.68 | 0.39-1.12 | 0.1661 |
| *Rs6019* | *F5* | 1.13 | 0.39-3.31 | 0.8205 |
| *Rs3753305* | *F5* | 1.36 | 0.83-2.22 | 0.2243 |
| *Rs5030390* | *ICAM1* | 1.59 | 0.57-4.45 | 0.3738 |
| *Rs281432* | *ICAM1* | 1.32 | 0.79-2.21 | 0.2934 |
| *Rs3093032* | *ICAM1* | 1.16 | 0.67-2.01 | 0.6048 |
| *Rs3093030* | *ICAM1* | 0.57 | 0.34-0.94 | 0.0270 |
| *Rs1799810* | *PROC* | 1.08 | 0.69-1.67 | 0.7466 |
| *Rs2069920* | *PROC* | 1.52 | 0.94-2.44 | 0.0878 |
| *Rs2069923* | *PROC* | 1.09 | 0.40-2.91 | 0.8770 |
| *Rs2069928* | *PROC* | 0.73 | 0.45-1.17 | 0.1948 |
| *Rs6113909* | *THBD* | 1.07 | 0.59-1.92 | 0.8324 |
| *Rs6082986* | *THBD* | 0.98 | 0.58-1.64 | 0.9343 |
| *Rs1962* | *THBD* | 1.25 | 0.73-2.14 | 0.4204 |
| *Rs3176123* | *THBD* | 0.58 | 0.35-0.96 | 0.0346 |
| *Rs3176119* | *THBD* | 0.85 | 0.34-2.09 | 0.7177 |
| *Rs3216183* | *THBD* | 0.81 | 0.46-1.42 | 0.4602 |
