# Supplementary material for: Combined Effects of Thrombosis Pathway Gene Variants Predict Cardiovascular Events
Source: PLoS Genet. 2007 Jul 27;3(7):e120. doi: 10.1371/journal.pgen.0030120 (PMC1934395; doi:10.1371/journal.pgen.0030120)
Supplement: Table S8 — Covariates: age at baseline, (sex, cohort), smoking, hypertension, TC/HDL, BMI, diabetes, and CRP. FINRISK-92 and FINRISK-97 cohorts combined for the analysis. Analysis performed according to dominant inheritance model; hazard ratios >1 show major allele as the risk allele. (12 KB DOC) [file pgen.0030120.st008.doc]

Supplementary Table 8: Association of the SNPs studied with incident ischemic stroke events in time-to-event analysis (covariates: age at baseline, (sex, cohort), smoking, hypertension, TC/HDL, BMI, diabetes, CRP) in men. FINRISK-92 and FINRISK-97 cohorts combined for the analysis. Analysis performed according to dominant inheritance model; hazard ratios >1 show major allele as the risk allele.

| SNP | Gene | Hazard Ratio | 95% Confidence  Interval | p-value |
| --- | --- | --- | --- | --- |
| ***Rs2420369*** | F5 | **0.74** | **0.38-1.46** | **0.3880** |
| ***Rs9332591*** | ***F5*** | **1.51** | **0.70-3.25** | **0.2957** |
| ***Rs6025*** | ***F5*** | **4.47** | **1.63-12.29** | **0.0037** |
| ***Rs7542281*** | ***F5*** | **1.75** | **0.83-3.71** | **0.1430** |
| ***Rs2269648*** | ***F5*** | **0.67** | **0.36-1.26** | **0.2175** |
| ***Rs5030347*** | ***ICAM1*** | **0.98** | **0.91-1.04** | **0.4640** |
| ***Rs5030341*** | ***ICAM1*** | **1.52** | **0.80-2.89** | **0.1968** |
| ***Rs5937*** | ***PROC*** | **1.09** | **0.62-1.92** | **0.7544** |
| ***Rs1401296*** | ***PROC*** | **1.17** | **0.64-2.13** | **0.6081** |
| ***Rs1042580*** | ***THBD*** | **0.92** | **0.49-1.72** | **0.7885** |
| ***Rs6048519*** | ***THBD*** | **1.07** | **0.56-2.04** | **0.8326** |
| *Rs970741* | *F5* | 0.83 | 0.44-1.57 | 0.5690 |
| *Rs6013* | *F5* | 1.60 | 0.67-3.82 | 0.2884 |
| *Rs9332640* | *F5* | 1.04 | 0.51-2.11 | 0.9190 |
| *Rs6030* | *F5* | 0.99 | 0.52-1.89 | 0.9785 |
| *Rs9332618* | *F5* | 0.82 | 0.47-1.44 | 0.4967 |
| *Rs9332695* | *F5* | 0.67 | 0.23-1.99 | 0.4742 |
| *Rs9332590* | *F5* | 1.24 | 0.71-2.16 | 0.4574 |
| *Rs6035* | *F5* | 1.59 | 0.58-4.34 | 0.3696 |
| *Rs9332575* | *F5* | 0.79 | 0.41-1.52 | 0.4803 |
| *Rs6019* | *F5* | 0.99 | 0.27-3.65 | 0.9835 |
| *Rs3753305* | *F5* | 1.30 | 0.68-2.49 | 0.4206 |
| *Rs5030390* | *ICAM1* | 1.68 | 0.52-5.38 | 0.3829 |
| *Rs281432* | *ICAM1* | 1.63 | 0.86-3.11 | 0.1355 |
| *Rs3093032* | *ICAM1* | 1.38 | 0.68-2.79 | 0.3728 |
| *Rs3093030* | *ICAM1* | 0.79 | 0.42-1.47 | 0.4527 |
| *Rs1799810* | *PROC* | 0.94 | 0.55-1.61 | 0.8272 |
| *Rs2069920* | *PROC* | 1.45 | 0.82-2.58 | 0.2039 |
| *Rs2069923* | *PROC* | 0.76 | 0.33-1.79 | 0.5304 |
| *Rs2069928* | *PROC* | 0.89 | 0.51-1.57 | 0.6982 |
| *Rs6113909* | *THBD* | 1.26 | 0.63-2.53 | 0.5089 |
| *Rs6082986* | *THBD* | 0.81 | 0.58-2.01 | 0.8091 |
| *Rs1962* | *THBD* | 2.02 | 0.95-4.28 | 0.0667 |
| *Rs3176123* | *THBD* | 0.64 | 0.64-0.33 | 0.1826 |
| *Rs3176119* | *THBD* | 0.86 | 0.29-2.53 | 0.7783 |
| *Rs3216183* | *THBD* | 0.67 | 0.32-1.40 | 0.2956 |
