# Supplementary material for: Combined Effects of Thrombosis Pathway Gene Variants Predict Cardiovascular Events
Source: PLoS Genet. 2007 Jul 27;3(7):e120. doi: 10.1371/journal.pgen.0030120 (PMC1934395; doi:10.1371/journal.pgen.0030120)
Supplement: Table S9 — Covariates: age at baseline, (sex, cohort), smoking, hypertension, TC/HDL, BMI, diabetes, and CRP. FINRISK-92 and FINRISK-97 cohorts combined for the analysis. Analysis performed according to dominant inheritance model; hazard ratios >1 show major allele as the risk allele. (12 KB DOC) [file pgen.0030120.st009.doc]

Supplementary Table 9: Association of the SNPs studied with incident ischemic stroke events in time-to-event analysis (covariates: age at baseline, (sex, cohort), smoking, hypertension, TC/HDL, BMI, diabetes, CRP) in women. FINRISK-92 and FINRISK-97 cohorts combined for the analysis. Analysis performed according to dominant inheritance model; hazard ratios >1 show major allele as the risk allele.

| SNP | Gene | Hazard Ratio | 95% Confidence  Interval | p-value |
| --- | --- | --- | --- | --- |
| ***Rs2420369*** | F5 | **2.52** | **0.47-13.62** | **0.2816** |
| ***Rs9332591*** | ***F5*** | **1.70** | **0.26-11.21** | **0.5832** |
| ***Rs6025*** | ***F5*** | **184.67** | **3.44-9921.76** | **0.0102** |
| ***Rs7542281*** | ***F5*** | **13.10** | **1.62-105.96** | **0.0159** |
| ***Rs2269648*** | ***F5*** | **2.42** | **0.38-15.24** | **0.3469** |
| ***Rs5030347*** | ***ICAM1*** | **0.99** | **0.83-1.19** | **0.9522** |
| ***Rs5030341*** | ***ICAM1*** | **1.70** | **0.31-9.45** | **0.5437** |
| ***Rs5937*** | ***PROC*** | **0.57** | **0.10-3.41** | **0.5373** |
| ***Rs1401296*** | ***PROC*** | **3.93** | **0.72-21.63** | **0.1156** |
| ***Rs1042580*** | ***THBD*** | **0.47** | **0.05-5.02** | **0.5302** |
| ***Rs6048519*** | ***THBD*** | **1.04** | **0.12-8.90** | **0.9721** |
| *Rs970741* | *F5* | 2.10 | 0.34-13.08 | 0.4259 |
| *Rs6013* | *F5* | 2.37 | 0.34-16.39 | 0.3809 |
| *Rs9332640* | *F5* | 1.00 | 0.10-10.41 | 0.9984 |
| *Rs6030* | *F5* | 4.46 | 0.73-27.22 | 0.1050 |
| *Rs9332618* | *F5* | 0.93 | 0.12-7.04 | 0.9455 |
| *Rs9332695* | *F5* | 1.26 | 0.15-10.45 | 0.8399 |
| *Rs9332590* | *F5* | 1.06 | 0.13-8.29 | 0.9589 |
| *Rs6035* | *F5* | 4.73 | 0.27-83.29 | 0.2886 |
| *Rs9332575* | *F5* | 0.12 | 0.02-0.61 | 0.0105 |
| *Rs6019* | *F5* | 21.71 | 1.43-329.12 | 0.0265 |
| *Rs3753305* | *F5* | 1.44 | 0.34-6.12 | 0.6194 |
| *Rs5030390* | *ICAM1* | 6.76 | 0.02-1966.29 | 0.5090 |
| *Rs281432* | *ICAM1* | 1.17 | 0.17-8.03 | 0.8767 |
| *Rs3093032* | *ICAM1* | 0.30 | 0.03-3.55 | 0.3378 |
| *Rs3093030* | *ICAM1* | 0.49 | 0.09-2.82 | 0.4249 |
| *Rs1799810* | *PROC* | 1.98 | 0.29-13.54 | 0.4881 |
| *Rs2069920* | *PROC* | 1.93 | 0.17-22.53 | 0.6014 |
| *Rs2069923* | *PROC* | 6.16 | 0.07-558.95 | 0.4293 |
| *Rs2069928* | *PROC* | 0.37 | 0.06-2.12 | 0.2618 |
| *Rs6113909* | *THBD* | 0.48 | 0.04-6.52 | 0.5834 |
| *Rs6082986* | *THBD* | 0.40 | 0.05-2.94 | 0.3675 |
| *Rs1962* | *THBD* | 0.47 | 0.05-4.14 | 0.4936 |
| *Rs3176123* | *THBD* | 0.69 | 0.10-4.64 | 0.7005 |
| *Rs3176119* | *THBD* | 19.89 | 2.88-137.59 | 0.0024 |
| *Rs3216183* | *THBD* | 1.23 | 0.13-11.33 | 0.8527 |
