# Supplementary material for: Combined Effects of Thrombosis Pathway Gene Variants Predict Cardiovascular Events
Source: PLoS Genet. 2007 Jul 27;3(7):e120. doi: 10.1371/journal.pgen.0030120 (PMC1934395; doi:10.1371/journal.pgen.0030120)
Supplement: Table S10 — Covariates: age at baseline, (sex, cohort), smoking, hypertension, TC/HDL, BMI, diabetes, and CRP. FINRISK-92 and FINRISK-97 cohorts combined for the analysis. Analysis performed according to dominant inheritance model; hazard ratios >1 show major allele as the risk allele. (12 KB DOC) [file pgen.0030120.st010.doc]

Supplementary Table 10: Association of the SNPs studied with incident cardiovascular (coronary or ischemic stroke) events in time-to-event analysis (covariates: age at baseline, (sex, cohort), smoking, hypertension, TC/HDL, BMI, diabetes, CRP). FINRISK-92 and FINRISK-97 cohorts combined for the analysis, which comprises both sexes. Analysis performed according to dominant inheritance model; hazard ratios >1 show major allele as the risk allele.

| SNP | Gene | Hazard ratio | 95% Confidence  Interval | p-value |
| --- | --- | --- | --- | --- |
| ***Rs2420369*** | F5 | **1.16** | **0.95-1.41** | **0.1585** |
| ***Rs9332591*** | ***F5*** | **0.88** | **1.11-1.41** | **0.3890** |
| ***Rs6025*** | ***F5*** | **1.48** | **0.97-2.25** | **0.0661** |
| ***Rs7542281*** | ***F5*** | **1.23** | **0.93-1.64** | **0.1539** |
| ***Rs2269648*** | ***F5*** | **1.10** | **0.90-1.34** | **0.3393** |
| ***Rs5030347*** | ***ICAM1*** | **0.97** | **0.96-0.99** | **0.0202** |
| ***Rs5030341*** | ***ICAM1*** | **1.16** | **0.95-1.43** | **0.1491** |
| ***Rs5937*** | ***PROC*** | **1.11** | **0.91-1.36** | **0.2838** |
| ***Rs1401296*** | ***PROC*** | **1.11** | **0.91-1.35** | **0.3188** |
| ***Rs1042580*** | ***THBD*** | **0.94** | **0.77-1.15** | **0.5506** |
| ***Rs6048519*** | ***THBD*** | **0.97** | **0.78-1.20** | **0.7646** |
| *Rs970741* | *F5* | 1.08 | 0.87-1.33 | 0.4896 |
| *Rs6013* | *F5* | 1.10 | 0.83-1.45 | 0.5008 |
| *Rs9332640* | *F5* | 1.21 | 0.98-1.48 | 0.0787 |
| *Rs6030* | *F5* | 1.14 | 0.94-1.40 | 0.1820 |
| *Rs9332618* | *F5* | 0.96 | 0.78-1.18 | 0.6767 |
| *Rs9332695* | *F5* | 0.76 | 0.54-1.06 | 0.1039 |
| *Rs9332590* | *F5* | 1.05 | 0.86-1.27 | 0.6372 |
| *Rs6035* | *F5* | 1.32 | 0.96-1.80 | 0.0850 |
| *Rs9332575* | *F5* | 0.98 | 0.77-1.25 | 0.8542 |
| *Rs6019* | *F5* | 1.15 | 0.76-1.76 | 0.5049 |
| *Rs3753305* | *F5* | 1.00 | 0.82-1.23 | 0.9898 |
| *Rs5030390* | *ICAM1* | 1.22 | 0.83-1.78 | 0.3144 |
| *Rs281432* | *ICAM1* | 1.09 | 0.88-1.36 | 0.4352 |
| *Rs3093032* | *ICAM1* | 1.08 | 0.86-1.35 | 0.5315 |
| *Rs3093030* | *ICAM1* | 0.94 | 0.77-1.16 | 0.5639 |
| *Rs1799810* | *PROC* | 1.07 | 0.88-1.29 | 0.5274 |
| *Rs2069920* | *PROC* | 0.98 | 0.80-1.20 | 0.8236 |
| *Rs2069923* | *PROC* | 1.05 | 0.69-1.58 | 0.8308 |
| *Rs2069928* | *PROC* | 0.90 | 0.74-1.09 | 0.2805 |
| *Rs6113909* | *THBD* | 0.96 | 0.78-1.17 | 0.6570 |
| *Rs6082986* | *THBD* | 0.93 | 0.76-1.13 | 0.4650 |
| *Rs1962* | *THBD* | 1.06 | 0.86-1.31 | 0.6091 |
| *Rs3176123* | *THBD* | 1.09 | 0.90-1.33 | 0.3734 |
| *Rs3176119* | *THBD* | 0.87 | 0.59-1.30 | 0.4967 |
| *Rs3216183* | *THBD* | 1.05 | 0.84-1.31 | 0.6830 |
