# Supplementary material for: Combined Effects of Thrombosis Pathway Gene Variants Predict Cardiovascular Events
Source: PLoS Genet. 2007 Jul 27;3(7):e120. doi: 10.1371/journal.pgen.0030120 (PMC1934395; doi:10.1371/journal.pgen.0030120)
Supplement: Table S11 — Covariates: age at baseline, (sex, cohort), smoking, hypertension, TC/HDL, BMI, diabetes, and CRP. FINRISK-92 and FINRISK-97 cohorts combined for the analysis. Analysis performed according to dominant inheritance model; hazard ratios >1 show major allele as the risk allele. (12 KB DOC) [file pgen.0030120.st011.doc]

Supplementary Table 11: Association of the SNPs studied with incident cardiovascular (coronary or ischemic stroke) events in time-to-event analysis (covariates: age at baseline, (sex, cohort), smoking, hypertension, TC/HDL, BMI, diabetes, CRP) in men. FINRISK-92 and FINRISK-97 cohorts combined for the analysis. Analysis performed according to dominant inheritance model; hazard ratios >1 show major allele as the risk allele.

| SNP | Gene | Hazard Ratio | 95% Confidence  Interval | p-value |
| --- | --- | --- | --- | --- |
| ***Rs2420369*** | F5 | **0.95** | **0.74-1.22** | **0.6767** |
| ***Rs9332591*** | ***F5*** | **1.14** | **0.85-1.54** | **0.3793** |
| ***Rs6025*** | ***F5*** | **1.55** | **0.92-2.61** | **0.1019** |
| ***Rs7542281*** | ***F5*** | **0.99** | **0.69-1.41** | **0.9412** |
| ***Rs2269648*** | ***F5*** | **1.04** | **0.81-1.32** | **0.7809** |
| ***Rs5030347*** | ***ICAM1*** | **0.99** | **0.96-1.02** | **0.3886** |
| ***Rs5030341*** | ***ICAM1*** | **1.13** | **0.88-1.44** | **0.3515** |
| ***Rs5937*** | ***PROC*** | **1.15** | **0.91-1.46** | **0.2473** |
| ***Rs1401296*** | ***PROC*** | **1.02** | **0.80-1.30** | **0.8809** |
| ***Rs1042580*** | ***THBD*** | **0.88** | **0.69-1.13** | **0.3241** |
| ***Rs6048519*** | ***THBD*** | **1.02** | **0.78-1.32** | **0.9034** |
| *Rs970741* | *F5* | 0.89 | 0.70-1.15 | 0.3761 |
| *Rs6013* | *F5* | 1.07 | 0.77-1.48 | 0.7027 |
| *Rs9332640* | *F5* | 1.11 | 0.86-1.43 | 0.4312 |
| *Rs6030* | *F5* | 0.98 | 0.77-1.25 | 0.8865 |
| *Rs9332618* | *F5* | 0.86 | 0.66-1.12 | 0.2629 |
| *Rs9332695* | *F5* | 0.75 | 0.50-1.11 | 0.1516 |
| *Rs9332590* | *F5* | 1.14 | 0.90-1.44 | 0.2908 |
| *Rs6035* | *F5* | 1.43 | 0.99-2.06 | 0.0587 |
| *Rs9332575* | *F5* | 0.92 | 0.68-1.24 | 0.5706 |
| *Rs6019* | *F5* | 1.19 | 0.71-2.02 | 0.5080 |
| *Rs3753305* | *F5* | 0.94 | 0.73-1.21 | 0.6140 |
| *Rs5030390* | *ICAM1* | 1.09 | 0.68-1.76 | 0.7135 |
| *Rs281432* | *ICAM1* | 1.12 | 0.86-1.46 | 0.3883 |
| *Rs3093032* | *ICAM1* | 1.14 | 0.87-1.50 | 0.3549 |
| *Rs3093030* | *ICAM1* | 0.94 | 0.73-1.22 | 0.6545 |
| *Rs1799810* | *PROC* | 1.01 | 0.80-1.28 | 0.9101 |
| *Rs2069920* | *PROC* | 1.01 | 0.79-1.28 | 0.9702 |
| *Rs2069923* | *PROC* | 0.97 | 0.61-1.55 | 0.8987 |
| *Rs2069928* | *PROC* | 0.98 | 0.77-1.24 | 0.8569 |
| *Rs6113909* | *THBD* | 0.90 | 0.70-1.17 | 0.4417 |
| *Rs6082986* | *THBD* | 0.88 | 0.69-1.12 | 0.2997 |
| *Rs1962* | *THBD* | 1.16 | 0.89-1.50 | 0.2770 |
| *Rs3176123* | *THBD* | 1.06 | 0.83-1.35 | 0.6370 |
| *Rs3176119* | *THBD* | 0.83 | 0.53-1.28 | 0.3941 |
| *Rs3216183* | *THBD* | 0.98 | 0.76-1.28 | 0.9010 |
