# Supplementary material for: Combined Effects of Thrombosis Pathway Gene Variants Predict Cardiovascular Events
Source: PLoS Genet. 2007 Jul 27;3(7):e120. doi: 10.1371/journal.pgen.0030120 (PMC1934395; doi:10.1371/journal.pgen.0030120)
Supplement: Table S12 — Covariates: age at baseline, (sex, cohort), smoking, hypertension, TC/HDL, BMI, diabetes, and CRP. FINRISK-92 and FINRISK-97 cohorts combined for the analysis. Analysis performed according to dominant inheritance model; hazard ratios >1 show major allele as the risk allele. (12 KB DOC) [file pgen.0030120.st012.doc]

Supplementary Table 12: Association of the SNPs studied with incident cardiovascular (coronary or ischemic stroke) events in time-to-event analysis (covariates: age at baseline, (sex, cohort), smoking, hypertension, TC/HDL, BMI, diabetes, CRP) in women. FINRISK-92 and FINRISK-97 cohorts combined for the analysis. Analysis performed according to dominant inheritance model; hazard ratios >1 show major allele as the risk allele.

| SNP | Gene | Hazard Ratio | 95% Confidence  Interval | p-value |
| --- | --- | --- | --- | --- |
| ***Rs2420369*** | F5 | **1.81** | **1.19-2.74** | **0.0055** |
| ***Rs9332591*** | ***F5*** | **1.05** | **0.66-1.68** | **0.8432** |
| ***Rs6025*** | ***F5*** | **1.48** | **0.60-3.69** | **0.3987** |
| ***Rs7542281*** | ***F5*** | **2.36** | **1.35-4.13** | **0.0026** |
| ***Rs2269648*** | ***F5*** | **1.52** | **0.98-2.35** | **0.0635** |
| ***Rs5030347*** | ***ICAM1*** | **0.95** | **0.91-1.03** | **0.0592** |
| ***Rs5030341*** | ***ICAM1*** | **1.43** | **0.93-2.20** | **0.1069** |
| ***Rs5937*** | ***PROC*** | **0.94** | **0.62-1.42** | **0.7630** |
| ***Rs1401296*** | ***PROC*** | **1.57** | **1.03-2.04** | **0.0354** |
| ***Rs1042580*** | ***THBD*** | **0.91** | **0.59-1.41** | **0.6814** |
| ***Rs6048519*** | ***THBD*** | **0.83** | **0.52-1.30** | **0.4081** |
| *Rs970741* | *F5* | 1.69 | 1.06-2.71 | 0.0292 |
| *Rs6013* | *F5* | 1.23 | 0.69-2.21 | 0.4833 |
| *Rs9332640* | *F5* | 1.57 | 1.01-2.43 | 0.0456 |
| *Rs6030* | *F5* | 1.67 | 1.09-2.55 | 0.0181 |
| *Rs9332618* | *F5* | 1.38 | 0.88-2.16 | 0.1670 |
| *Rs9332695* | *F5* | 0.75 | 0.50-1.11 | 0.1516 |
| *Rs9332590* | *F5* | 0.69 | 0.46-1.04 | 0.0780 |
| *Rs6035* | *F5* | 0.88 | 0.46-1.69 | 0.7032 |
| *Rs9332575* | *F5* | 1.09 | 0.81-1.48 | 0.5706 |
| *Rs6019* | *F5* | 1.32 | 0.58-3.02 | 0.5094 |
| *Rs3753305* | *F5* | 1.15 | 0.78-1.69 | 0.4891 |
| *Rs5030390* | *ICAM1* | 1.85 | 0.79-4.33 | 0.1582 |
| *Rs281432* | *ICAM1* | 1.13 | 0.70-1.82 | 0.6267 |
| *Rs3093032* | *ICAM1* | 0.91 | 0.58-1.42 | 0.6673 |
| *Rs3093030* | *ICAM1* | 0.94 | 0.73-1.22 | 0.6545 |
| *Rs1799810* | *PROC* | 1.13 | 0.77-1.68 | 0.5315 |
| *Rs2069920* | *PROC* | 0.94 | 0.60-1.48 | 0.7954 |
| *Rs2069923* | *PROC* | 1.26 | 0.52-3.05 | 0.6130 |
| *Rs2069928* | *PROC* | 0.66 | 0.44-0.99 | 0.0422 |
| *Rs6113909* | *THBD* | 0.84 | 0.55-1.29 | 0.4314 |
| *Rs6082986* | *THBD* | 0.86 | 0.57-1.31 | 0.4764 |
| *Rs1962* | *THBD* | 1.03 | 0.65-1.62 | 0.9053 |
| *Rs3176123* | *THBD* | 0.81 | 0.54-1.21 | 0.3009 |
| *Rs3176119* | *THBD* | 0.73 | 0.32.1.67 | 0.4549 |
| *Rs3216183* | *THBD* | 1.12 | 0.71-1.76 | 0.6375 |
