# Supplementary material for: Combined Effects of Thrombosis Pathway Gene Variants Predict Cardiovascular Events
Source: PLoS Genet. 2007 Jul 27;3(7):e120. doi: 10.1371/journal.pgen.0030120 (PMC1934395; doi:10.1371/journal.pgen.0030120)
Supplement: Table S13 — Covariates: age at baseline, (sex, cohort), smoking, hypertension, TC/HDL, BMI, diabetes, and CRP). FINRISK-92 and FINRISK-97 cohorts combined for the analysis. Analysis performed according to dominant inheritance model; hazard ratios >1 show major allele as the risk allele. (12 KB DOC) [file pgen.0030120.st013.doc]

Supplementary Table 13: Association of the SNPs studied with total mortality in time-to-event analysis (covariates: age at baseline, (sex, cohort), smoking, hypertension, TC/HDL, BMI, diabetes, CRP). FINRISK-92 and FINRISK-97 cohorts combined for the analysis, which comprises both sexes. Analysis performed according to dominant inheritance model; hazard ratios >1 show major allele as the risk allele.

| SNP | Gene | Hazard Ratio | 95% Confidence  Interval | p-value |
| --- | --- | --- | --- | --- |
| ***Rs2420369*** | ***F5*** | **1.08** | **0.89-1.30** | **0.4352** |
| ***Rs9332591*** | ***F5*** | **0.93** | **0.76-1.14** | **0.4937** |
| ***Rs6025*** | ***F5*** | **0.58** | **0.33-0.99** | **0.0503** |
| ***Rs7542281*** | ***F5*** | **1.02** | **0.78-1.34** | **0.8754** |
| ***Rs2269648*** | ***F5*** | **0.99** | **0.82-1.17** | **0.8996** |
| ***Rs5030347*** | ***ICAM1*** | **0.97** | **0.95-0.99** | **0.0016** |
| ***Rs5030341*** | ***ICAM1*** | **1.04** | **0.86-1.26** | **0.6661** |
| ***Rs5937*** | ***PROC*** | **1.06** | **0.88-1.26** | **0.5561** |
| ***Rs1401296*** | ***PROC*** | **1.08** | **0.90-1.29** | **0.3981** |
| ***Rs1042580*** | ***THBD*** | **1.06** | **0.89-1.26** | **0.5420** |
| ***Rs6048519*** | ***THBD*** | **0.93** | **0.76-1.13** | **0.4654** |
| *Rs970741* | *F5* | 1.02 | 0.84-1.23 | 0.8713 |
| *Rs6013* | *F5* | 1.13 | 0.86-1.49 | 0.3706 |
| *Rs9332640* | *F5* | 1.04 | 0.85-1.27 | 0.7092 |
| *Rs6030* | *F5* | 1.07 | 0.88-1.29 | 0.4839 |
| *Rs9332618* | *F5* | 1.15 | 0.94-1.40 | 0.1727 |
| *Rs9332695* | *F5* | 1.18 | 0.82-1.68 | 0.3694 |
| *Rs9332590* | *F5* | 0.92 | 0.77-1.11 | 0.3961 |
| *Rs6035* | *F5* | 0.78 | 0.60-1.02 | 0.0737 |
| *Rs9332575* | *F5* | 1.16 | 0.93-1.44 | 0.1808 |
| *Rs6019* | *F5* | 1.43 | 0.90-2.29 | 0.1332 |
| *Rs3753305* | *F5* | 1.13 | 0.94-1.36 | 0.1926 |
| *Rs5030390* | *ICAM1* | 0.95 | 0.69-1.30 | 0.7395 |
| *Rs281432* | *ICAM1* | 1.00 | 0.82-1.23 | 0.9658 |
| *Rs3093032* | *ICAM1* | 1.06 | 0.85-1.31 | 0.6215 |
| *Rs3093030* | *ICAM1* | 1.04 | 0.86-1.25 | 0.7027 |
| *Rs1799810* | *PROC* | 1.07 | 0.89-1.28 | 0.4732 |
| *Rs2069920* | *PROC* | 0.94 | 0.79-1.14 | 0.5505 |
| *Rs2069923* | *PROC* | 1.16 | 0.78-1.73 | 0.4697 |
| *Rs2069928* | *PROC* | 0.87 | 0.72-1.04 | 0.1180 |
| *Rs6113909* | *THBD* | 1.04 | 0.86-1.25 | 0.6854 |
| *Rs6082986* | *THBD* | 1.06 | 0.89-1.27 | 0.4969 |
| *Rs1962* | *THBD* | 0.96 | 0.79-1.16 | 0.6859 |
| *Rs3176123* | *THBD* | 0.96 | 0.80-1.15 | 0.6467 |
| *Rs3176119* | *THBD* | 0.83 | 0.97-1.34 | 0.8302 |
| *Rs3216183* | *THBD* | 1.01 | 0.83-1.23 | 0.9537 |
