# Supplementary material for: Combined Effects of Thrombosis Pathway Gene Variants Predict Cardiovascular Events
Source: PLoS Genet. 2007 Jul 27;3(7):e120. doi: 10.1371/journal.pgen.0030120 (PMC1934395; doi:10.1371/journal.pgen.0030120)
Supplement: Table S14 — Covariates: age at baseline, (sex, cohort), smoking, hypertension, TC/HDL, BMI, diabetes, and CRP. FINRISK-92 and FINRISK-97 cohorts combined for the analysis. Analysis performed according to dominant inheritance model; hazard ratios >1 show major allele as the risk allele. (12 KB DOC) [file pgen.0030120.st014.doc]

Supplementary Table 14: Association of the SNPs studied with incident cardiovascular (coronary or ischemic stroke) events in time-to-event analysis (covariates: age at baseline, (sex, cohort), smoking, hypertension, TC/HDL, BMI, diabetes, CRP) in men. FINRISK-92 and FINRISK-97 cohorts combined for the analysis. Analysis performed according to dominant inheritance model; hazard ratios >1 show major allele as the risk allele.

| SNP | Gene | Hazard Ratio | 95% Confidence  Interval | p-value |
| --- | --- | --- | --- | --- |
| ***Rs2420369*** | ***F5*** | **0.93** | **0.74-1.17** | **0.5150** |
| ***Rs9332591*** | ***F5*** | **1.04** | **0.81-1.35** | **0.7540** |
| ***Rs6025*** | ***F5*** | **0.65** | **0.35-1.23** | **0.1839** |
| ***Rs7542281*** | ***F5*** | **0.95** | **0.68-1.33** | **0.7690** |
| ***Rs2269648*** | ***F5*** | **0.98** | **0.79-1.21** | **0.8281** |
| ***Rs5030347*** | ***ICAM1*** | **0.96** | **0.95-0.99** | **0.0032** |
| ***Rs5030341*** | ***ICAM1*** | **1.02** | **0.81-1.28** | **0.8666** |
| ***Rs5937*** | ***PROC*** | **1.08** | **0.87-1.34** | **0.4708** |
| ***Rs1401296*** | ***PROC*** | **1.04** | **0.84-1.29** | **0.7379** |
| ***Rs1042580*** | ***THBD*** | **1.07** | **0.86-1.32** | **0.5640** |
| ***Rs6048519*** | ***THBD*** | **0.95** | **0.75-1.20** | **0.6509** |
| *Rs970741* | *F5* | 1.00 | 0.80-1.26 | 0.9880 |
| *Rs6013* | *F5* | 1.09 | 0.79-1.52 | 0.5985 |
| *Rs9332640* | *F5* | 0.93 | 0.73-1.18 | 0.5509 |
| *Rs6030* | *F5* | 1.04 | 0.83-1.30 | 0.7345 |
| *Rs9332618* | *F5* | 1.13 | 0.89-1.44 | 0.3178 |
| *Rs9332695* | *F5* | 1.13 | 0.74-1.73 | 0.5809 |
| *Rs9332590* | *F5* | 0.96 | 0.77-1.19 | 0.6975 |
| *Rs6035* | *F5* | 0.76 | 0.56-1.03 | 0.0769 |
| *Rs9332575* | *F5* | 1.12 | 0.86-1.46 | 0.4003 |
| *Rs6019* | *F5* | 1.27 | 0.75-2.16 | 0.3760 |
| *Rs3753305* | *F5* | 1.08 | 0.86-1.35 | 0.5290 |
| *Rs5030390* | *ICAM1* | 0.85 | 0.59-1.23 | 0.3843 |
| *Rs281432* | *ICAM1* | 1.06 | 0.84-1.34 | 0.6496 |
| *Rs3093032* | *ICAM1* | 0.98 | 0.76-1.26 | 0.8627 |
| *Rs3093030* | *ICAM1* | 1.10 | 0.88-1.38 | 0.4135 |
| *Rs1799810* | *PROC* | 1.07 | 0.86-1.33 | 0.5500 |
| *Rs2069920* | *PROC* | 0.94 | 0.75-1.18 | 0.5901 |
| *Rs2069923* | *PROC* | 1.34 | 0.79-2.28 | 0.2760 |
| *Rs2069928* | *PROC* | 0.89 | 0.71-1.10 | 0.2820 |
| *Rs6113909* | *THBD* | 1.05 | 0.84-1.32 | 0.6491 |
| *Rs6082986* | *THBD* | 1.07 | 0.86-1.33 | 0.5308 |
| *Rs1962* | *THBD* | 0.97 | 0.78-1.22 | 0.8209 |
| *Rs3176123* | *THBD* | 0.98 | 0.79-1.21 | 0.8316 |
| *Rs3176119* | *THBD* | 1.07 | 0.73-1.58 | 0.7205 |
| *Rs3216183* | *THBD* | 0.96 | 0.76-1.22 | 0.7522 |
