# Supplementary material for: Combined Effects of Thrombosis Pathway Gene Variants Predict Cardiovascular Events
Source: PLoS Genet. 2007 Jul 27;3(7):e120. doi: 10.1371/journal.pgen.0030120 (PMC1934395; doi:10.1371/journal.pgen.0030120)
Supplement: Table S15 — Covariates: age at baseline, (sex, cohort), smoking, hypertension, TC/HDL, BMI, diabetes, and CRP). FINRISK-92 and FINRISK-97 cohorts combined for the analysis. Analysis performed according to dominant inheritance model; hazard ratios >1 show major allele as the risk allele. (12 KB DOC) [file pgen.0030120.st015.doc]

Supplementary Table 15: Association of the SNPs studied with total mortality in time-to-event analysis (covariates: age at baseline, (sex, cohort), smoking, hypertension, TC/HDL, BMI, diabetes, CRP) in women. FINRISK-92 and FINRISK-97 cohorts combined for the analysis. Analysis performed according to dominant inheritance model; hazard ratios >1 show major allele as the risk allele.

| SNP | Gene | Hazard Ratio | 95% Confidence  Interval | p-value |
| --- | --- | --- | --- | --- |
| ***Rs2420369*** | ***F5*** | **1.55** | **0.99-1.52** | **0.0580** |
| ***Rs9332591*** | ***F5*** | **0.76** | **0.52-1.10** | **0.1458** |
| ***Rs6025*** | ***F5*** | **0.41** | **0.14-1.21** | **0.1060** |
| ***Rs7542281*** | ***F5*** | **1.25** | **0.76-2.07** | **0.3801** |
| ***Rs2269648*** | ***F5*** | **1.14** | **0.80-1.63** | **0.4766** |
| ***Rs5030347*** | ***ICAM1*** | **0.98** | **0.95-1.02** | **0.3919** |
| ***Rs5030341*** | ***ICAM1*** | **1.07** | **0.74-1.54** | **0.7321** |
| ***Rs5937*** | ***PROC*** | **0.92** | **0.65-1.31** | **0.6483** |
| ***Rs1401296*** | ***PROC*** | **1.10** | **0.76-1.57** | **0.6156** |
| ***Rs1042580*** | ***THBD*** | **0.99** | **0.70-1.41** | **0.9560** |
| ***Rs6048519*** | ***THBD*** | **1.01** | **0.70-1.47** | **0.9708** |
| *Rs970741* | F5 | 1.05 | 0.73-1.51 | 0.8029 |
| *Rs6013* | *F5* | 1.14 | 0.69-1.88 | 0.6101 |
| *Rs9332640* | *F5* | 1.43 | 0.98-2.09 | 0.0606 |
| *Rs6030* | *F5* | 1.15 | 0.81-1.63 | 0.4493 |
| *Rs9332618* | *F5* | 1.25 | 0.84-1.87 | 0.2663 |
| *Rs9332695* | *F5* | 1.28 | 0.62-2.59 | 0.5167 |
| *Rs9332590* | *F5* | 0.83 | 0.58-1.19 | 0.2988 |
| *Rs6035* | *F5* | 0.78 | 0.44-1.38 | 0.3937 |
| *Rs9332575* | *F5* | 0.74 | 0.51-1.09 | 0.1283 |
| *Rs6019* | *F5* | 1.98 | 0.71-5.50 | 0.1902 |
| *Rs3753305* | *F5* | 1.33 | 0.93-1.88 | 0.1151 |
| *Rs5030390* | *ICAM1* | 1.48 | 0.72-3.06 | 0.2846 |
| *Rs281432* | *ICAM1* | 0.86 | 0.58-1.27 | 0.5813 |
| *Rs3093032* | *ICAM1* | 1.23 | 0.81-1.88 | 0.3267 |
| *Rs3093030* | *ICAM1* | 0.92 | 0.65-1.32 | 0.6559 |
| *Rs1799810* | *PROC* | 1.06 | 0.74-1.50 | 0.7625 |
| *Rs2069920* | *PROC* | 0.90 | 0.61-1.33 | 0.6063 |
| *Rs2069923* | *PROC* | 0.95 | 0.46-1.96 | 0.8785 |
| *Rs2069928* | *PROC* | 0.89 | 0.62-1.27 | 0.4432 |
| *Rs6113909* | *THBD* | 0.99 | 0.70-1.43 | 0.9913 |
| *Rs6082986* | *THBD* | 0.99 | 0.71-1.40 | 0.9741 |
| *Rs1962* | *THBD* | 0.97 | 0.65-1.43 | 0.8648 |
| *Rs3176123* | *THBD* | 1.14 | 0.80-1.62 | 0.4653 |
| *Rs3176119* | *THBD* | 0.68 | 0.35-1.33 | 0.2680 |
| *Rs3216183* | *THBD* | 1.04 | 0.71-1.52 | 0.8489 |
