# Supplementary material for: Combined Effects of Thrombosis Pathway Gene Variants Predict Cardiovascular Events
Source: PLoS Genet. 2007 Jul 27;3(7):e120. doi: 10.1371/journal.pgen.0030120 (PMC1934395; doi:10.1371/journal.pgen.0030120)
Supplement: Table S16 — (12 KB DOC) [file pgen.0030120.st016.doc]

Supplementary Table 16: SNPs not chosen for stage 2 analyses based on the classification-trees and showing p-values <0.05 in the analysis for combined study cohorts analyzed in the separate cohorts FINRISK-92 and FINRISK-97.

|  |  |  | FINRISK-92 |  |  | FINRISK-97 |  |  |
| --- | --- | --- | --- | --- | --- | --- | --- | --- |
| SNP | Gene | Group ^a^ | HR ^b^ | 95% CI ^c^ | p-value | HR ^b^ | 95% CI  ^c^ | p-value |
| *Rs9332575* | F5 | STR F | 1.59 | 0.53-4.81 | 0.4105 | 0.81 | 0.39-1.68 | 0.5743 |
| *Rs9332640* | *F5* | CVD F | 1.12 | 0.88-1.42 | 0.1098 | 0.87 | 0.56-1.88 | 0.5477 |
| *Rs970741* ^d^ | *F5* | CVD F | 0.93 | 0.61-1.41 | 0.7270 | 0.87 | 0.64-1.19 | 0.3854 |
| *Rs6030* | *F5* | CVD F | 1.04 | 0.70-1.55 | 0.8540 | 0.99 | 0.73-1.35 | 0.9671 |
| *Rs6035* | *F5* | CVD M | 2.32 | 1.11-4.85 | 0.0252 | 0.97 | 0.76-1.81 | 0.4667 |
| *Rs6035* | *F5* | STR all | 2.61 | 1.28-5.30 | 0.0081 | 1.21 | 0.50-2.96 | 0.6708 |
| *Rs3093030* | *ICAM1* | STR all | 0.93 | 0.54-1.62 | 0.7995 | 1.13 | 0.83-1.53 | 0.4368 |
| *Rs2069928* | *PROC* | CVD F | 0.94 | 0.65-1.36 | 0.7517 | 1.01 | 0.74-1.38 | 0.9441 |
| *Rs3176123* | *THBD* | STR all | 0.56 | 0.26-1.12 | 0.1410 | 1.01 | 0.53-1.75 | 0.9023 |

^a^ STR: Incident ischemic stroke; CVD: Incident ischemic stroke or coronary event; F: Females; M: Males; All: Males and females ^b^ HR: Hazard ratio ^c^ CI: Confidence interval ^d^ *Rs970741* was chosen for stage 2 analyses based on classification-trees but was excluded based on LD with SNP *rs2420369.*
